# Trophic strategies of intertidal foraminifera explored with single‐cell microbiome metabarcoding and morphological methods: What is on the menu?

**DOI:** 10.1002/ece3.9437

**Published:** 2022-11-15

**Authors:** Magali Schweizer, Thierry Jauffrais, Constance Choquel, Vona Méléder, Sophie Quinchard, Emmanuelle Geslin

**Affiliations:** ^1^ UMR 6112 LPG, Laboratoire de Planétologie et Géosciences, Univ Angers, Nantes Université, Le Mans Université CNRS Angers France; ^2^ UMR 9220 ENTROPIE, Ifremer, IRD, Univ Nouvelle‐Calédonie, Univ La Réunion CNRS Noumea New Caledonia; ^3^ Department of Geology Lund University Lund Sweden; ^4^ UR 2160, ISOMer, Institut des Substances et Organismes de la Mer Nantes Université Nantes France

**Keywords:** kleptoplasty, microbiome, mudflat, protist, SSU rDNA, trophic behavior

## Abstract

In mudflats, interactions and transfers of nutrients and secondary metabolites may drive ecosystems and biodiversity. Foraminifera have complex trophic strategies as they often rely on bacteria and eukaryotes or on potential symbionts for carbon and nitrogen resources. The capacity of these protists to use a wide range of adaptive mechanisms requires clarifying the relationships between them and their microbial associates. Here, we investigate the interactions of three foraminiferal species with nearby organisms in situ, by coupling molecular (cloning/Sanger and high‐throughput sequencing) and direct counting and morphological identification with microscopy. This coupling allows the identification of the organisms found in or around three foraminiferal species through molecular tools combined with a direct counting of foraminifera and diatoms present in situ through microscopy methods. Depending on foraminiferal species, and in addition to diatom biomass, diatom frustule shape, size and species are key factors driving the abundance and diversity of foraminifera in mudflat habitats. Three different trophic strategies were deduced for the foraminifera investigated in this study: *Ammonia* sp. T6 has an opportunistic strategy and is feeding on bacteria, nematoda, fungi, and diatoms when abundant; *Elphidium oceanense* is feeding mainly on diatoms, mixed with other preys when they are less abundant; and *Haynesina germanica* is feeding almost solely on medium‐large pennate diatoms. Although there are limitations due to the lack of species coverage in DNA sequence databases and to the difficulty to compare morphological and molecular data, this study highlights the relevance of combining molecular with morphological tools to study trophic interactions and microbiome communities of protists at the single‐cell scale.

## Graphical abstract

This study focuses on trophic interactions between foraminifera and their preys in tidal flats. Morphological data (counts and taxonomy) of foraminifera and diatoms are compared with sediment and microbiome DNA sequences of 16S and 18S to infer the diet of the three most abundant species of foraminifera.

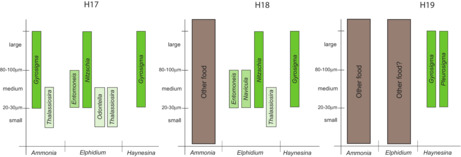

## 1 INTRODUCTION

Intertidal mudflats host abundant and diverse microbial communities that play major roles in primary production, food web, biogeochemical cycles, and sediment stabilization (Cesbron et al., 2016; Lebreton et al., 2019; Lubarsky et al., 2010; MacIntyre et al., 1996; Miller et al., 1996). Among these communities, the microphytobenthos (MPB) is composed of an assemblage of benthic photosynthetic microalgae and cyanobacteria often dominated by diatoms (MacIntyre et al., 1996; Méléder et al., 2007). It is a major contributor of mudflats primary production and a food source for heterotrophs (Blanchard et al., 2001; Miller et al., 1996; Underwood & Kromkamp, 1999). Furthermore, microbial eukaryotes/prokaryotes interactions are essential to marine ecosystems as they facilitate nutrient recycling, photosynthetic activity, and secondary metabolite production (Amin et al., 2012). Microphytic species are in perpetual interactions with bacteria (Van Colen et al., 2014) and unicellular eukaryotes, such as foraminifera, which often rely on both bacteria and microalgae as a source for nutrition (e.g., Bird et al., 2018; Enge et al., 2011; Haynert et al., 2020; Lintner et al., 2020; Mojtahid et al., 2011; Nomaki et al., 2005, 2006; Pascal et al., 2009; Witte et al., 2003; Wukovits et al., 2017) or for potential symbionts (e.g., Bernhard, 2003; Bernhard et al., 2006, 2018; Bird et al., 2017; Lee et al., 1988; Pawlowski et al., 2001; Prazeres et al., 2017). These interactions and transfers of nutrients and secondary metabolites (LeKieffre et al., 2017) may thus drive mudflat ecosystems and biodiversity.

All kinds of trophic strategies can be found in unicellular eukaryotes, from photoautotrophy to mixotrophy and obligate heterotrophy (e.g., Chakraborty et al., 2017; Stefanidou et al., 2018; Stoecker et al., 2017). Inside this broad group, the phylum Foraminifera (Retaria, Rhizaria) encompasses different feeding strategies such as heterotrophy or mixotrophy, but autotrophy was never observed. Heterotrophic strategies in foraminifera include selective and indiscriminate grazing (Moodley et al., 2002; Nomaki et al., 2008; Pascal et al., 2009), uptake of dissolved organic matter (DeLaca et al., 1981), passive suspension feeding (Cedhagen, 1988), predation (Bird et al., 2018; Bowser et al., 1985; Dupuy et al., 2010; Suhr et al., 2008), or parasitism (Alexander & DeLaca, 1987; Cedhagen, 1994). Mixotrophic strategies comprise symbioses with prokaryotes and eukaryotes (e.g., Bird et al., 2017, 2018; Lee et al., 1991; Pawlowski et al., 2001) and kleptoplasty (Jauffrais et al., 2016; LeKieffre et al., 2018; Lopez, 1979). The presence of prokaryotic symbionts has been described in benthic foraminifera from oxygen‐depleted environments (Bernhard, 2003; Bernhard et al., 2000, 2006; Nomaki et al., 2014; Tsuchiya et al., 2015), but also in well‐oxygenated sediments (Richardson & Rützler, 1999), intertidal environments (Koho et al., 2018; Salonen et al., 2019) and in the plankton realm (Bird et al., 2017). Eukaryotic symbiosis in foraminifera is well developed in oligotrophic environments such as tropical neritic or planktonic habitats (e.g., Bird et al., 2018; Hallock, 1999; Lee, 2006; Pawlowski et al., 2001; Prazeres et al., 2017). Kleptoplasty is a symbiotic phenomenon whereby plastids, notably chloroplasts from algae, are sequestered by host organisms (Clark et al., 1990). Several genera of foraminifera from photic and aphotic zones have been found to perform it with diatom chloroplasts (e.g., Bernhard & Bowser, 1999; Jauffrais et al., 2016, 2018; Lee et al., 1988; Lopez, 1979; Tsuchiya et al., 2015). Efficient photosynthesis has been proven only in kleptoplastic foraminifera from photic zones (Jauffrais et al., 2016; Jauffrais, LeKieffre, Schweizer, Geslin, et al., 2019; Jauffrais, LeKieffre, Schweizer, Jesus, et al., 2019; Jesus et al., 2021; LeKieffre et al., 2018; Lopez, 1979).

Among all trophic studies concerning foraminifera, the ones focusing on in situ feeding strategies are scarce (Glock, Wukovits, et al., 2019; Goldstein, 1999; Haynert et al., 2020; Nomaki et al., 2005; Tsuchiya et al., 2018; Witte et al., 2003). Nevertheless, a growing number of studies using molecular approaches to characterize the preys and endobionts of foraminifera in situ have been published in recent years (Bird et al., 2017, 2018; Chronopoulou et al., 2019; Jauffrais, LeKieffre, Schweizer, Geslin, et al., 2019; Jauffrais, LeKieffre, Schweizer, Jesus, et al., 2019; Pillet et al., 2011; Prazeres et al., 2017; Salonen et al., 2019; Schmidt et al., 2016; Tsuchiya et al., 2015).

Within the three main calcitic genera found in European mudflats, *Ammonia* is thought to be omnivorous, feeding on organic detritus, bacteria, microalgae, and meiofauna (Dupuy et al., 2010; Mojtahid et al., 2011; Pascal et al., 2009; Wukovits et al., 2018). Among species of *Elphidium* living in mudflats, *Elphidium oceanense* (d'Orbigny in Fornasini, 1904), *Elphidium selseyense* (Heron‐Allen and Earland, 1911), and *Elphidium williamsoni* Haynes, 1973, are kleptoplastic (Jauffrais et al., 2018; Jauffrais, LeKieffre, Schweizer, Jesus, et al., 2019; Jesus et al., 2021; Lopez, 1979; Pillet et al., 2011). Nevertheless, it has not been proven yet that the kleptoplasts are photosynthetically active in *E. oceanense* and *E. selseyense*. *Haynesina germanica* (Ehrenberg, 1840) has been shown to feed on large diatoms (Austin et al., 2005) and to be a photosynthetically active kleptoplastic species (Jauffrais et al., 2016; Jesus et al., 2021; LeKieffre et al., 2018; Lopez, 1979). A recent study using a metabarcoding approach confirmed the omnivorous diet of *Ammonia* and the kleptoplastic activity of *E. selseyense* and *H. germanica* (Chronopoulou et al., 2019).

The capacity of foraminifera to use a wide range of adaptive mechanisms is exemplified by denitrification (Choquel et al., 2021; Glock, Roy, et al., 2019; Piña‐Ochoa et al., 2010; Risgaard‐Petersen et al., 2006; Woehle et al., 2018), prokaryotic (Bernhard et al., 2018; Bird et al., 2017) or microalgal (Hallock, 1999; Prazeres et al., 2017) symbioses and kleptoplasty (Jauffrais et al., 2018; Lopez, 1979). Investigating these mechanisms requires clarifying the relationships between foraminifera and their microbial associates. Improving our knowledge on foraminiferal trophic interactions would allow to better understand this understudied group and its role in the ecosystem functioning and in the biogeochemical cycles.

In the present study, we combine molecular (cloning/Sanger sequencing and high‐throughput sequencing or HTS) with morphological (granulometric measurements and optical microscopy observations) approaches to investigate the identity of organisms interacting with foraminifera in situ. Both approaches have pros and cons. Microscopy allows to count specimens, but is more limited for species identification, whereas eDNA is more precise for species identification, but has only semiquantitative resolution. Here, we define the microbiome as the nonforaminiferal DNA sequenced from foraminifera, which can originate from symbionts, commensals, parasites, decomposers, or preys. Three to five specimens of three foraminiferal species are collected from three sites in the Bourgneuf mudflat (France). Single foraminifer extractions are used to sequence bacteria and chloroplasts with the 16S rDNA marker and eukaryotes with the 18S rDNA marker to investigate organisms interacted with the foraminifera. Samples are sequenced using HTS to get an overview of the diversity of taxa found associated with foraminifera. In addition, we used a cloning/Sanger sequencing on one sample/species/site to gather the most abundant and representative sequences present in the different foraminiferal microbiomes. We did so because this approach allows obtaining longer sequences than HTS, and therefore a taxonomic assignment of much higher quality and resolution. In parallel, optical microscopy observations are performed to count foraminifera and diatoms from fixed volumes of sediment to get an estimation of the densities of both groups in situ at the time of sampling. We expect that combining these methodologies, which is new for foraminiferal studies, will improve our results by getting advantages of eDNA for species identification, and microscopy for density estimation.

## 2 MATERIAL AND METHODS

### 2.1 Sampling site and granulometry

**FIGURE 1:**
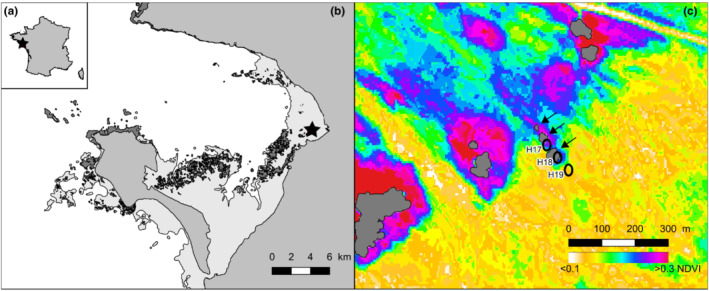
Localization of the three sampling stations in the Bay of Bourgneuf: (a) France with a star indicating the studied region, (b) Bay of Bourgneuf with a star indicating the sampling area, (c) the stations H17, H18, and H19 near to oyster reefs (indicated by arrows). In (b) light gray surfaces indicate the lower level of spring tide and dark gray surfaces the rocky areas, including oyster reefs. The intertidal zone in (c) is covered with an NDVI map retrieved from a Spot image (15/09/12, 10:54 GMT).

Sediment samples were collected on the 1st of October 2015 in the Bay of Bourgneuf, situated south of the Loire estuary on the French west coast (Figure 1a) with a large intertidal mudflat (100 km^2^). Three stations were chosen close to a natural oyster reef and at ~50 m apart from each other (Figure 1b,c): H17 (47°01′33.15″N 2°00′21.58″W) between two oyster reefs (~15 m from each reef), H18 (47°01′31.91″N 2°00′20.06″W) near the southern oyster reef (~5 m from the reef), and H19 (47°01′30.68″N 2°00′18.52″W) the furthest from oyster reefs (~50 m apart), in the bare mudflat.

For the granulometric analysis, the superficial sediment layer (~ first centimeter) was scrapped with a spoon in the three stations and *brought* back to the laboratory in a cooling box and frozen on arrival. For each station, 1 g of sediment was prepared and analyzed through liquid dispersion with a laser diffraction particle size analyzer (Malvern Mastersizer 3000E, Malvern Instruments) at the UMR 6112 LPG (University of Angers). This analysis allowed to define sediment grain size by the relative abundance (% volume) of silt (Ø < 63 μm) and sand (63 < Ø < 2000 μm) according to the Udden‐Wentworth's scale.

### 2.2 NDVI and microphytobenthic assemblages in the first millimeters

To retrieve the MPB biomass for each station, a SPOT image was analyzed following Méléder et al. (2003) and Echappé et al. (2018). This image was acquired from the sampled area by the SPOT 7 satellite, with 6 m of spatial resolution, on the 12th of September 2015 at 10:54 GMT, that is, 1:28 after the maximum low tide, which was 1.30 m (Echappé et al., 2018). Reflectance data from each pixel of the image were translated into NDVI (Normalized Difference Vegetation Index) values (Figure 1c), used as a proxy of the MPB biomass due to the chlorophyll *a* absorption (Benyoucef et al., 2014; Brito et al., 2013; Méléder et al., 2003), and averaged over an area of ~600 m^2^ (i.e., 20 pixels) around each sampling station and are compared (ANOVA and Tukey‐test).

The first millimeters of sediment with biofilm (~10 ml) were scraped using a clean spoon for the three stations. Samples were kept in cooling boxes during the few hours of transportation and were stored at −20°C back in the laboratory until processing. The organic matter, including microorganisms, was isolated from the sediment following a method adapted from Blanchard et al. (1988) by Méléder et al. (2007) using Ludox® HS‐40 colloidal silica (see Méléder et al., 2007 for details) and was collected and microscopically observed to estimate the occurrence of other microalgal taxa than diatoms. Then, samples were rinsed with distilled water and definitive slides were made after oxidation of the remaining organic matter (with H_2_O_2_ for a day, and then for 2 h at 450°C) to observe clean diatom frustules mounted in a high‐resolution diatom mountant (Naphrax, Brunel Microscopes Ltd). Morphospecies were identified using an Olympus Provis AX70 (magnification ×50) and following previous reference works (Ribeiro, 2010; Witkowski et al., 2000). In addition, samples of cleaned frustules were mounted on cover slips fixed to metallic supports and coated with platinum (thickness 2 nm) to be examined by a Scanning Electron Microscope (SEM) JEOL JSM 7600F (Institut des Matériaux Jean Rouxel [IMN], University of Nantes). For qualitative analyses of the morphospecies composition of MPB assemblages, a total of ~300 diatom frustules were counted in each sample to determine specific abundances. The total fields of view observed at ×50 were also counted. When the number of ~300 frustules could not be reached due to the dilution of cells within the samples, at least 250 fields of views were observed. In parallel, a biometric analysis was done on few specimens (*n* > 3) of the more abundant morphospecies to estimate their lengths and widths.

### 2.3 Foraminiferal assemblages in the first centimeter

In each of the three stations, three replicates were cored at one meter apart (H17.1, H17.2, H17.3; H18.1, H18.2, H18.3; H19.1, H19.2, and H19.3). The first centimeter of the sediment core (diameter 8.2 cm, standardized volume of 50 cm^3^ after Schönfeld et al., 2012) was used to assess living (Rose Bengal [RB] stained) foraminiferal assemblages. The samples were sliced and stained immediately after collection in 96% ethanol with 2 g/L RB, following the FOBIMO protocol (Schönfeld et al., 2012). The slices were then washed and sieved, and the 150–315 μm fraction was examined under a stereomicroscope. As the density of living foraminifera was high, the samples were split two to eight times to reduce the picking time while still getting a minimum of 300 individuals per replicate. Foraminifera were recognized on morphological criteria identified by combined molecular and morphological studies. For *Ammonia*, two species were distinguished based on the morphological characters described by Richirt et al. (2019). Species of *Elphidium* and *Haynesina* were named according to the study of Darling et al. (2016). SEM images of the representative taxa were taken with a Zeiss EVO LS10 (SCIAM, University of Angers) at low vacuum (50 Pa) without coating. Only the main species (>5%) were analyzed. Statistical analyses were performed with the R software (R Core Team, 2017) to compare the different foraminiferal population densities among stations by using a Kruskal–Wallis test for nonparametric data. When the results were significantly different, a Dunn's post hoc pairwise test (Dunn, 1964) was applied to identify which density differs from the others.

### 2.4 Foraminiferal and microbiome molecular identification

#### 2.4.1 Sampling and DNA extraction

The superficial sediment layer (~ first centimeter) was scraped with a sterile spoon in the three stations and *brought* back to the laboratory in a cooling box. In the laboratory, samples were kept at 4°C in darkness until processed. The next day the sediment was sieved (>150 μm) with artificial seawater (ASW, Red Sea Salt in milliQ water, salinity 34) and examined in ASW under a stereomicroscope. Live foraminifera (i.e., naturally colored cytoplasm inside the test and an empty last chamber) were carefully collected and placed in Petri dishes with ASW to check for vitality after a few hours (Schweizer et al., 2005). Active specimens (reticulopodial activity and movement) were collected, cleaned with a fine brush previously soaked in ethanol, and rinsed several times with clean ASW. A total of 45 foraminifera, belonging to *Ammonia* sp. T6, *E. oceanense* and *H. germanica*, the three most common species of foraminifera in the Bay of Bourgneuf (see Section 3.3), were isolated and individually placed in DOC (Deoxycholate) buffer for total DNA extraction (Pawlowski, 2000). Five specimens of each species were sampled in each of the three stations. Additional superficial sediment was scraped directly with 50 ml Falcon™ tubes from sites H17, H18, and H19 and immediately placed in the cooling box for the journey back to the laboratory. There, it was stored at −20°C until DNA was extracted from 10 g of sediment from each site with the DNeasy PowerSoil Kit (Qiagen) according to the manufacturer's instructions.

#### 2.4.2 High‐throughput sequencing

The primers 515f and 806r were used to amplify a ~250 bp fragment in the V4 region of 16S rDNA (Caporaso et al., 2011) and the primers 1380f and 1510r to amplify a ~160 bp fragment in the V9 region of 18S rDNA (Amaral‐Zettler et al., 2009). Primers were modified at the 5′ end to include Illumina adapters for the downstream sequencing. A first amplification with AccuPrime Taq HiFi (Fisher Scientific) and a volume of 50 μl was performed for both 16S and 18S regions and duplicated to minimize the intrasample variance and obtain enough amplified material. The amplification conditions were a first denaturation step at 94°C (1 min), followed by 40 cycles at 94°C (30 s), 50°C (30 s), and 72°C (1 min), and a final elongation at 72°C for 3 min. Negative controls (no added DNA) were performed in parallel. Amplicons, including negative controls, were purified by magnetic beads, and a second amplification was performed to incorporate Illumina adapters and tags with a combinatorial dual indexing of eight nucleotides to distinguish the samples. The following conditions were applied: a first denaturation step at 94°C (1 min), followed by 12 cycles at 94°C (1 min), 55°C (1 min), and 68°C (1 min), and a final elongation at 68°C for 10 min. Amplicons were purified as previously described and quantified with the QuantIT PicoGreen dsDNA Assay Kit (ThermoFisher Scientific). All the amplicons were pooled in equimolar concentrations, and the concentration of the pool was monitored with quantitative PCR (KAPA SYBR FAST, Merck). Amplicon libraries were mixed with 10% PhiX and sequenced with MiSeq reagent kit v2 500 cycles in two separate runs (18S and 16S) at the IRHS in Angers.

18S and 16S fastq files were processed separately. Raw reads were de‐multiplexed to samples with DADA2 v.1.6.0 (Callahan et al., 2016). MiSeq overhangs and primers were removed with Cutadapt v.3.5 (Martin, 2011). Paired‐end reads were assembled, quality‐filtered, aligned, checked for chimeras, clustered, and taxonomically assigned in Mothur v.1.44.3 (Schloss et al., 2009), following the MiSeq SOP (https://mothur.org/wiki/miseq_sop/). Reads were aligned against the SILVA database v.132 (Quast et al., 2013), and chimeric sequences were removed with UCHIME (Edgar et al., 2011). Clustering into operational taxonomic units (OTUs) was done using the 97% similarity sequence cutoff. Reads were taxonomically assigned with SILVA v.132 for 16S and with PR^2^ v.4.12.0 (Guillou et al., 2012) for 18S. The resulting tables (OTUs numbers per sample and taxonomic identity of OTUs) were then combined in R (R Core Team, 2017) and analyzed in Excel (Microsoft). OTUs with <10 reads and more than 10% of reads belonging to negative controls were removed.

#### 2.4.3 Sanger sequencing

To better identify the taxa found in the foraminifera, longer DNA fragments from a subset of foraminiferal specimens (one per species per site) were amplified, cloned, and sequenced with the Sanger method. Extractions were amplified with three different sets of primers to amplify fragments of the SSU rDNA gene for different groups: foraminifera, prokaryotes/chloroplasts (16S), and eukaryotes (18S). For foraminifera, taxon‐specific primers s14F3‐J2 and s14F1‐N6 (Darling et al., 2016; Pawlowski, 2000) were used with two rows of PCR following the protocol described in Darling et al. (2016). The amplified region (~500 bp) is situated at the 3′ end of the SSU rDNA, in the 18S V9 region, and is used for foraminiferal barcoding (Pawlowski & Holzmann, 2014). 16S rDNA and 18S were also amplified from the same DNA extractions following the protocol described in Jauffrais, LeKieffre, Schweizer, Geslin, et al. (2019). Two separate amplifications were performed on each extraction through two primer sets (Pillet et al., 2011), one targeting prokaryotic and chloroplastic 16S rDNA (PLA491F‐OXY1313R) and the other one targeting eukaryotic nuclear 18S rDNA (DiatSSUF‐DiatSSUR). The primers PLA491F and OXY1313R amplify a ~800 bp fragment of the 16S rDNA. They were designed to detect a broad range of chloroplasts, including embryophytes and green algae and can also amplify certain bacteria (Jauffrais, LeKieffre, Schweizer, Jesus, et al., 2019). Primers DiatSSUF and DiatSSUR amplify a ~830 bp region in the middle of the 18S rDNA. These primers, first designed as diatom specific (Pillet et al., 2011), amplify in fact a wide range of eukaryotes (Jauffrais, LeKieffre, Schweizer, Geslin, et al., 2019; Jauffrais, LeKieffre, Schweizer, Jesus, et al., 2019).

Nine specimens of foraminifera were analyzed, but only eight were successfully sequenced for foraminiferal DNA, 406 clones from nine extractions for 16S rDNA and 275 clones from eight extractions were sequenced for 18S. Positive amplifications of foraminiferal SSU were directly sequenced, whereas positive amplifications of 16S and 18S were separately purified with the High Pure PCR Purification Kit (Roche Diagnostics) and cloned using the pGEM®‐T Easy Vector System (Promega). Foraminiferal amplifications and clones were sequenced with the Sanger method (GATC Biotech, Cologne). Chromatograms of the sequences were checked by eye and cut manually when they became less accurate. For taxonomic identification, DNA sequences were compared with BLAST (Basic Local Alignment Search Tool, blast.ncbi.nlm.nih.gov, Altschul et al., 1997) and SILVA ACT (Alignment, Classification and Tree service, www.arb‐silva.de/aligner/, Pruesse et al., 2012).

In addition, the diatomaceous 18S rDNA sequences were placed with a representative selection of sequences belonging to diatoms taken from GenBank and aligned with MUSCLE (Edgar, 2004) implemented in Seaview v.4 (Gouy et al., 2010). Four subsets were prepared with the GenBank sequences most closely related to the studied sequences. Molecular phylogenetic trees were built with the PHYML program (Guindon & Gascuel, 2003) implemented in Seaview v.4, choosing the GTR (General Time Reversible) evolutionary model (Tavaré, 1986) and the approximate likelihood ratio test (aLRT) for branch support estimation (Anisimova & Gascuel, 2006).

To further analyze the Sanger sequences retrieved from the nine foraminiferal specimens, a microbiome interspecies comparison was done using the Gephi software (http://gephi.github.io/; Bastian et al., 2009) on 16S and 18S rDNA data. Gephi is a software often used in biology allowing the visualization of network (Jacomy et al., 2014; Serive et al., 2017). The DNA network analysis associated rDNA data (16S and 18S) extracted from the three studied foraminiferal species; that is, the software grouped the DNA data in communities sharing a common foraminiferal species. Circles with a high diameter represent foraminiferal species, while circles with a smaller diameter represent diatoms and other taxa found in their cytoplasm and identified with 16S and 18S rDNA. We used the ForceAtlas2 algorithm of the software to carry out the network analysis; it is a force‐directed layout where nodes repulse each other while edges attract their nodes. The final network helped to interpret intuitively the data through a community‐based analysis network (Jacomy et al., 2014). For clarity, 16S and 18S rDNA data mainly found in either *Ammonia* sp., *Haynesina germanica* or *Elphidium oceanense* were presented using colors identical to the ones of the foraminifera in which they were mainly encountered.

## 3 RESULTS

### 3.1 Granulometry of the sampled stations

**FIGURE 2:**
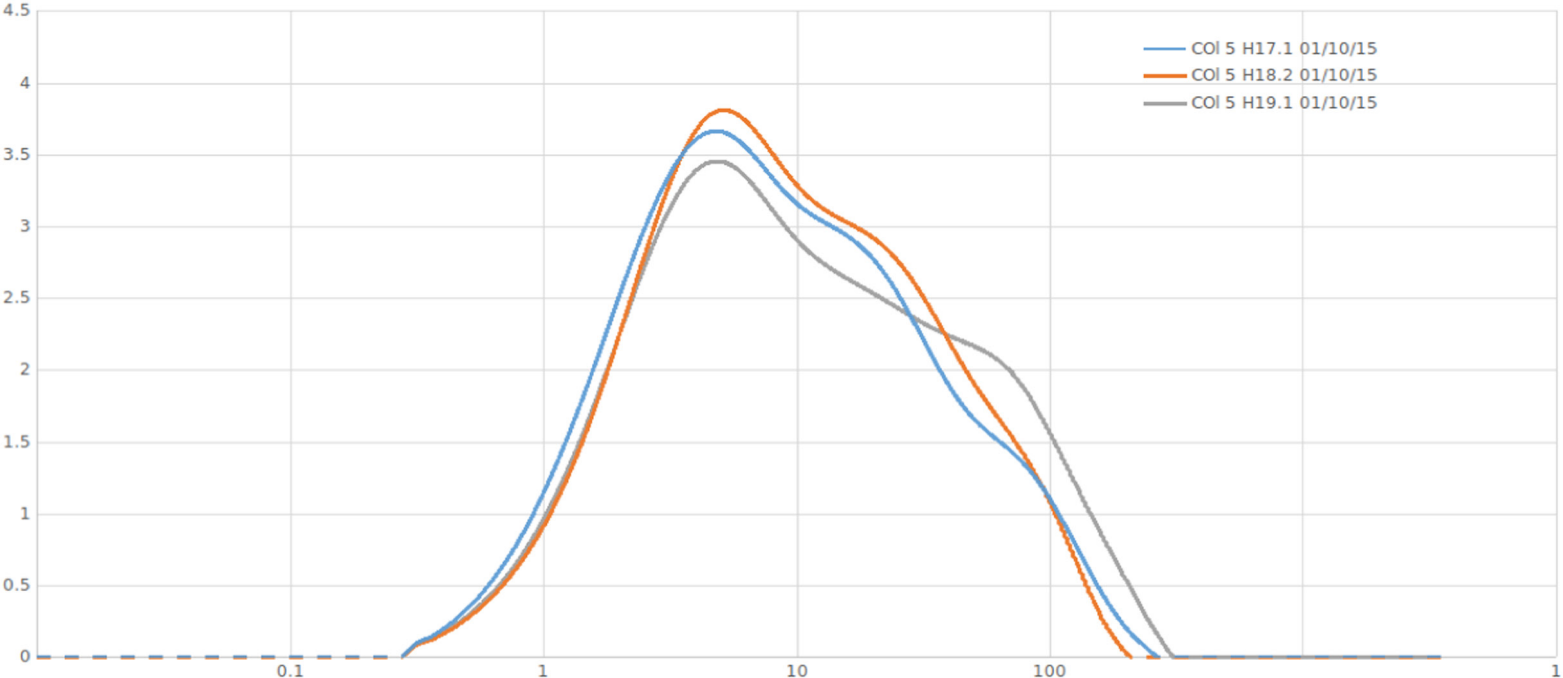
Granulometric characterization of the sediment in H17, H18, and H19 with comparison of particle diameter (μm) against class weight (%).

Stations H17 and H18 have a similar distribution of grain sizes, whereas station H19 is slightly coarser (Figure 2). Stations H17 and H18 contained, respectively, 90.6% and 91.2% of mud and 9.4% and 8.8% of sand, while station H19 contained 85.6% of mud and 14.4% of sand.

### 3.2 NDVI and microphytobenthic assemblages in the first millimeter

The biomass map (Figure 1c) showed NDVI values on the intertidal mudflat ranked as expected from 0.1 (no biomass) to 0.3 (maximum of MPB biomass) (for comparison, see Echappé et al., 2018; Méléder et al., 2003). NDVI mean values, calculated for H17, H18, and H19 were, respectively, 0.27 ± 0.01 (*n* = 18), 0.26 ± 0.009 (*n* = 10), and 0.15 ± 0.009 (*n* = 25), indicating that H19 was the station with the lowest biomass whereas both H17 and H18 were similar (*n* = 3; ANOVA: *F* = 39.14, *p* ≤ .001; Tukey‐test: *t* = 1.02, *p* = .76 between H17 and H19, *t* = 10.3 and *t* = 11.31, both *p* ≤ .001 between H19 and, respectively, H18 and H17). In the three sediment samples analyzed by microscopic observations, the biomass at the surface was mainly due to MPB assemblages only composed of diatoms. The densities of diatoms confirmed the trend observed with NDVI: a decrease from H17 to H19 with 2.56 individuals per field of view counted for H17, 1.46 for H18, and only 0.67 for H19 (Table 1).

**TABLE 1:** Numbers of diatoms counted for the three stations

	H17	H18	H19
Number of counted fields (×50)	158	235	270
Number of empty fields	19	97	141
*Amphora* spp.	2	0	6
***Cocconeis* spp.**	5	4	10
***Cymatosira belgica***	59	52	8
*Diploneis* sp.	1	0	0
*Eunotogramma dubium*	2	4	2
*Gyrosigma balticum*	6	0	0
***Gyrosigma wansbeckii***	44	22	0
*Gyrosigma* sp.	7	0	1
***Navicula phyllepta***	10	32	14
*Navicula* sp. 1	5	0	0
*Navicula* sp. 2	2	0	0
***Navicula spartinetensis***	24	91	24
*Nitzschia sigma*	20	4	0
*Nitzschia* sp.	3	0	0
*Plagiogrammopsis vanheurckii*	8	4	4
***Plagiotropis seriata***	92	0	0
*Plagiotropis vanheurckii*	2	12	0
***Planothidium delicatulum***	2	10	45
*Pleurosigma aestuari*	0	3	0
*Pleurosigma angulatum*	3	1	0
*Raphoneis* sp.	1	3	1
***Staurophora salina***	21	27	0
***Thalassiosira* spp. and *Odontella* spp.**	63	53	42
*Tryblionella apiculata*	0	0	4
Other diatom species	17	22	21
Total of specimens	399	344	182

*Note*: Taxa representing >75% of the assemblage are in bold.

**FIGURE 3:**
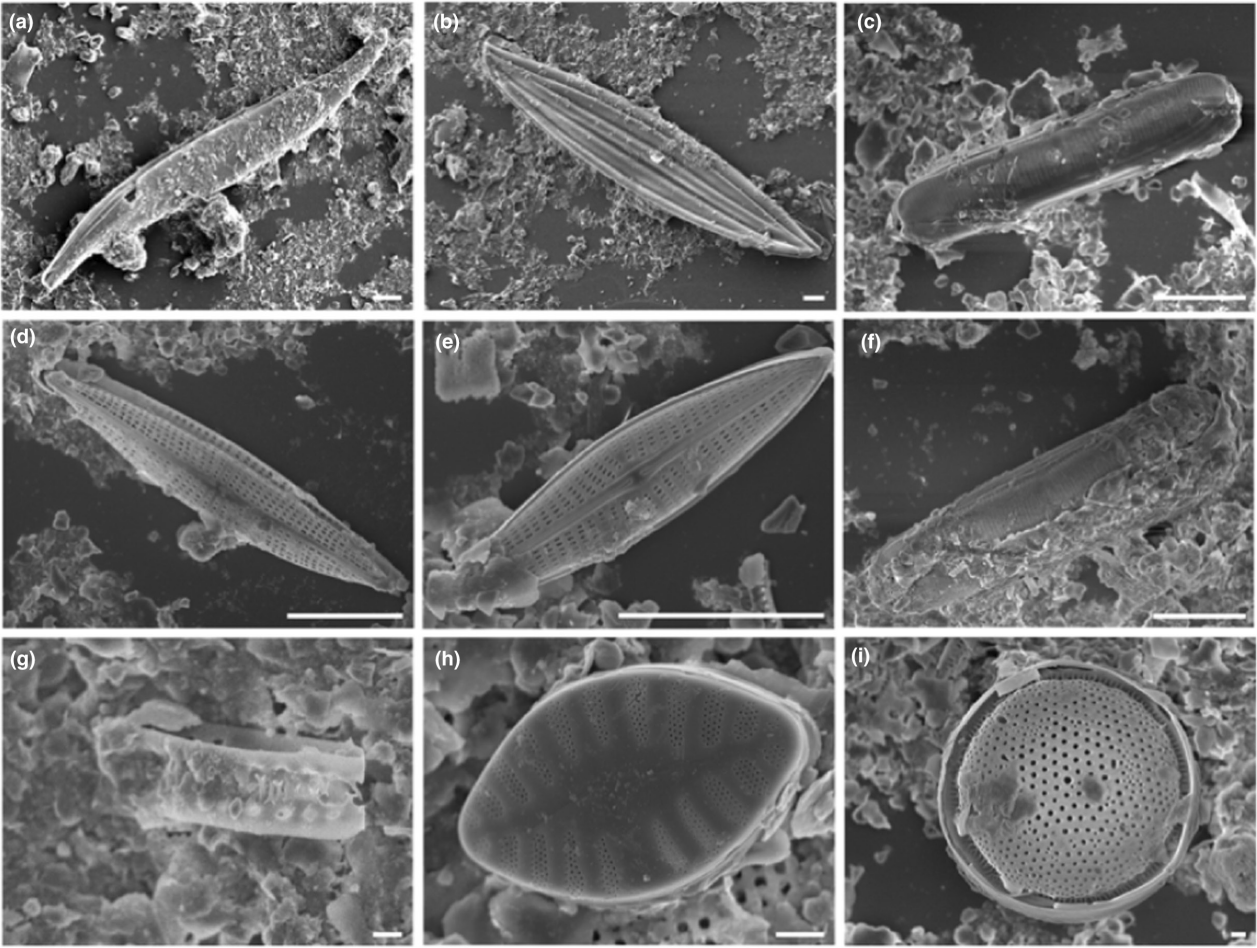
SEM images of frustules collected in situ from major diatom morphospecies (>75% of the assemblage) sampled during the study (imaged by B. Jesus, ISOMer). Station H17: (a) *Gyrosigma wansbeckii*, (b) *Plagiotropis seriata*, (c) *Staurophora salina*; Station H18: (d) *Navicula spartinetensis*, (e) *Navicula phyllepta*, (f) *Staurophora salina*, (g) *Cymatosira belgica*, (h) *Planothidium delicatulum*, and (i) *Thalassiosira* sp. Scale bar: 10 μm, except (g–i): 1 μm.

**FIGURE 4:**
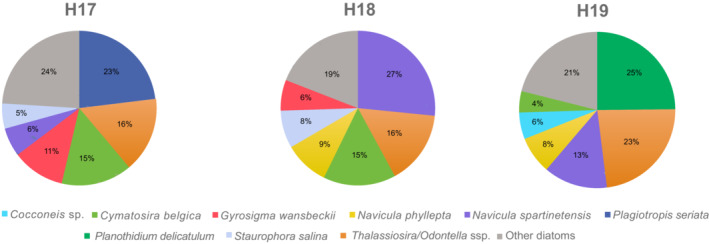
Relative frequency distribution of the counted diatoms for each station. Taxa representing >75% of the assemblage individually represented in each site.

**FIGURE 5:**
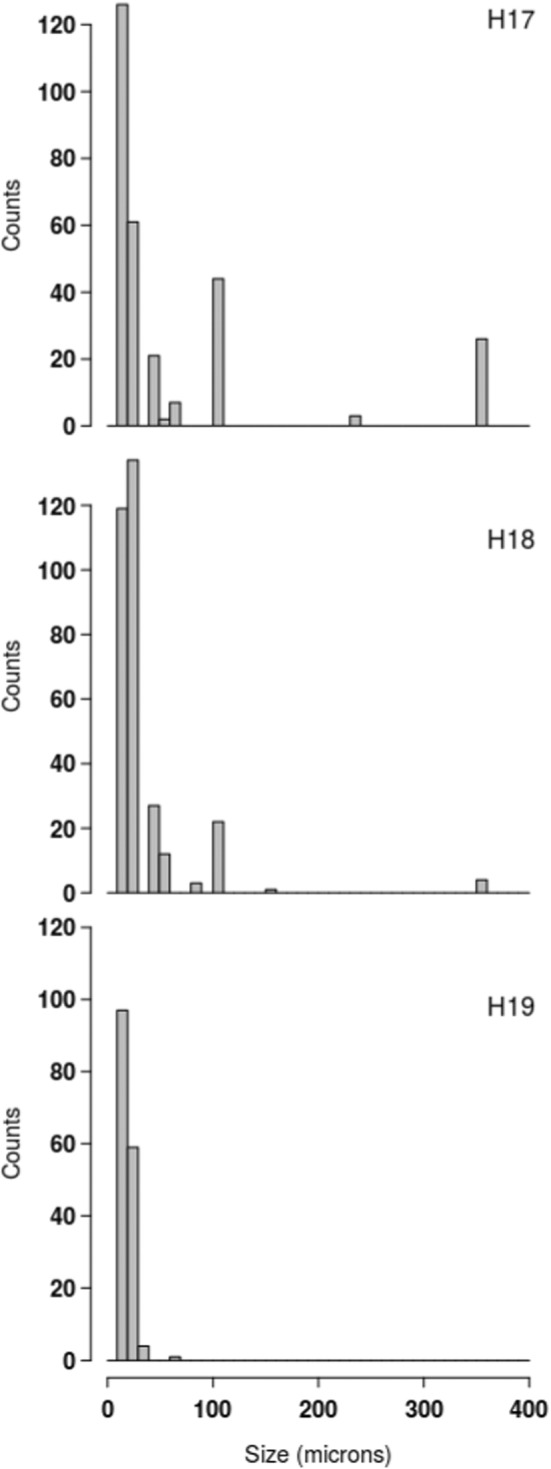
Numbers of individuals per size category for all diatoms collected in each station.

There were nine morphological taxa of diatoms commonly identified in this study: *Cocconeis* sp., *Cymatosira belgica* Grunow in Van Heurck, 1881, *Gyrosigma wansbeckii* (Donkin) Cleve, 1894, *Navicula phyllepta* Kützing, 1844, *Navicula spartinetensis* Sullivan & Reimer, 1975, *Plagiotropis seriata* (Cleve) Kuntze, 1898, *Planothidium delicatulum* (Kützing) Round & Bukhtiyarova, 1996, *Staurophora salina* (W. Smith) Mereschkowsky, 1903, and *Thalassiosira*/*Odontella* spp. (Figure 3). For this later group, both genera could not be distinguished with photonic microscopy (similar size and shape), it was possible only through SEM observations (Figure 3i). Nevertheless, the SEM observations were not numerous enough for abundance estimations. At each station, six of the nine morphospecies dominated the diatom assemblage, representing >75% of the total individuals (Figure 4). *Plagiotropis seriata*, a very long pennate diatom (Figure 3b, Table 2), was the most abundant morphospecies in H17 and was only found at this station. *Navicula spartinetensis*, a smaller pennate (Figure 3d, Table 2) dominated H18 and *P. delicatulum* (Figure 3h, Table 2) H19, respectively, although they were found in lower numbers in the other stations (Table 1). Centric diatoms were the second major group for the three stations with *Thalassiosira* and *Odontella* spp. (Figure 3i, Table 2). *Cymatosira belgica* was the third most abundant morphospecies in H17 and H18 and the sixth in H19 (Tables 1 and 2; Figure 4). This species is a small colonial diatom (Figure 3g, Table 2), forming chains of few specimens linked by valvar bifurcate linking spines, which increase the width by four or five. Most of the diatoms sampled in this study have a size below 100 μm (Table 2; Figure 5). However, in H17 and H18, there were also several diatoms larger than 100 μm (e.g., *P. seriata* and *G. wansbeckii*) and even close to 400 μm in some cases (*Nitzschia sigma* and *Gyrosigma balticum*), but it was not the case in H19 with only small (e.g., *P. delicatulum*, *C. belgica*, and *Cocconeis*) and medium (*N. spartinetensis* and *N. phyllepta*) morphospecies (Table 2; Figure 5).

**TABLE 2:** Dimensions of the diatom frustules found in the Bay of Bourgneuf

	Length (μm)	Width (μm)	Growth form
*Amphora* sp.	<25		Haptobenthic^a^
*Cocconeis* sp.	<25		Haptobenthic
*Cymatosira belgica*	11.8 ± 2.3 (*n* = 6)	3.3 ± 0.17 (*n* = 6)	Thycoplankton^b^
*Diploneis* sp.	<25		Epipelic^c^
*Eunotogramma dubium*	11.4*	3.7*	Epipsammic^d^
*Gyrosigma* sp.	59.9 ± 5.4 (*n* = 2)	8.1 ± 1.0 (*n* = 2)	Epipelic
*Gyrosigma balticum*	352.9*	29.4*	Epipelic
*Gyrosigma wansbeckii*	109.9 ± 8.1 (*n* = 13)	18.1 ± 2.2 (*n* = 13)	Epipelic
*Navicula* sp. 1	<25		Epipelic
*Navicula* sp. 2	<25		Epipelic
*Navicula phyllepta*	22.3 ± 6.4 (*n* = 8)	4.3 ± 0.72 (*n* = 8)	Epipelic
*Navicula spartinetensis*	24.6 ± 4.7 (*n* = 14)	4.9 ± 0.53 (*n* = 14)	Epipelic
*Nitzschia* sp.	<25		Epipelic
*Nitzschia sigma*	350.2 ± 34.8 (*n* = 10)	10.5 ± 2.2 (*n* = 10)	Epipelic
*Plagiogrammopsis vanheurckii*	22.5**	4**	Thycoplankton
*Plagiotropis seriata*	167.8 ± 6.7 (*n* = 7)	34.9 ± 3.8 (*n* = 7)	Epipelic
*Plagiotropis vanheurckii*	56.6*	10.2*	Epipelic
*Planothidium delicatulum*	<25		Epipsammic
*Pleurosigma aestuari*	80**	17**	Epipelic
*Pleurosigma angulatum*	239.8 ± 30.2 (*n* = 2)	48.6 ± 0.37 (*n* = 2)	Epipelic
*Raphoneis* sp.	<25		Epipsammic
*Staurophora salina*	42.3 ± 6.3 (*n* = 16)	10.0 ± 2.3 (*n* = 16)	Epipelic
*Thalassiosira* spp. and *Odontella* spp.	18.6 ± 7.5 (*n* = 6)		Thycoplankton
*Tryblionella apiculata*	31.2*	7.0*	Epipelic
Other diatoms	<25		

*Note*: Biometry from this study and *Ribeiro (2010); **Méléder (2003). Growth form from Hernández Fariñas et al. (2017), Poulíčková et al. (2008), Ribeiro (2010) and Round (1981).

^a^ Haptobenthic: taxa that live closely attached to, or growing on, solid submerged surfaces. In this case, it applies to genera with species that live in different hard substrata (e.g., sand grains, rocks, plants) and, therefore, may include species with different growth forms (e.g. epipsamic^d^).

^b^ Thycoplankton: taxa that have a benthic/pelagic cycling regulated by coincidental turbulence.

^c^ Epipelic: large motile diatoms, that can move freely between sediment particles and typically form biofilms.

^d^ Epipsammic: organisms that live in close association (attached or free living) with individual sediment particles, usually sand grains.

### 3.3 Foraminiferal assemblages in the first centimeter

**FIGURE 6:**
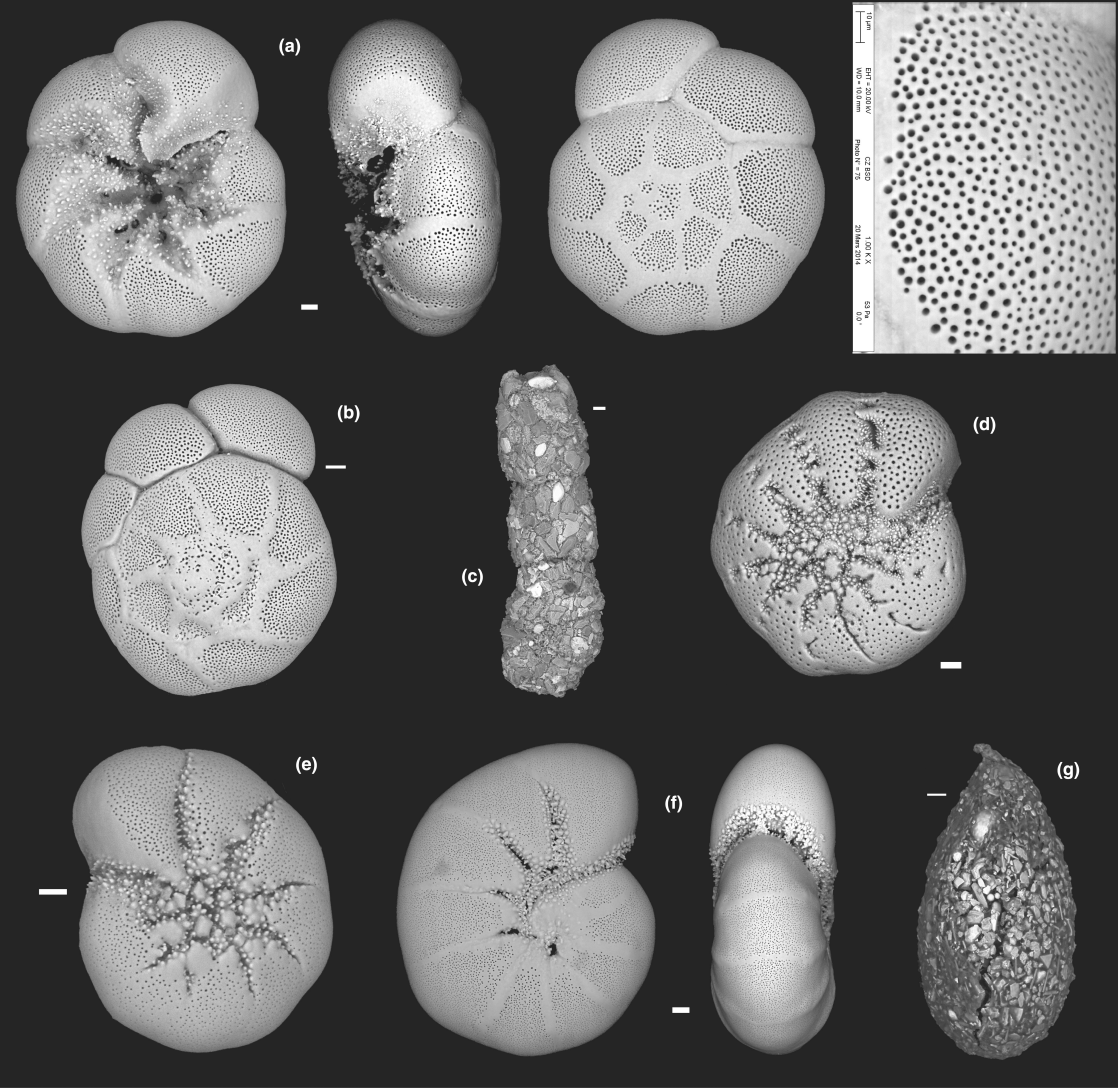
SEM images of the shelled foraminifera commonly found in the Bay of Bourgneuf (imaged by R. Mallet, SCIAM and M. Schweizer, LPG). Scale bar = 100 μm (except pores of *Ammonia* [a], scale bar = 10 μm): (a) *Ammonia* sp. T6 (isolate Bn009): umbilical, apertural and spiral sides, pores; (b) *Ammonia* sp. T1: spiral side; (c) *Ammotium salsum*; (d) *Elphidium oceanense* (isolate Bn122): lateral side; (e) *Elphidium selseyense* (isolate Bn162): lateral side; (f) *Haynesina germanica* (isolate Bn022): lateral and apertural sides; (g) *Psammophaga* sp.

Seven taxa were recognized morphologically in the living foraminiferal assemblage (Figure 6): *Ammonia* sp. T1 and *Ammonia* sp. T6 (Bird et al., 2020; Hayward et al., 2004; Richirt et al., 2019), *Ammotium salsum* (Cushman and Brönnimann, 1948), *Elphidium oceanense*, *E. selseyense*, *Haynesina germanica*, and *Psammophaga* sp. (Table 3). The most abundant species (>5%) were *Ammonia* sp. T6, *E. oceanense*, and *H. germanica* (Figure 6a,d,f).

**TABLE 3:** Numbers of foraminifera counted for the three replicates of each station

	H17.1	H17.2	H17.3	H18.1	H18.2	H18.3	H19.1	H19.2	H19.3
***Ammonia* sp. T6**	564	109	416	712	760	924	420	49	372
*Ammonia* sp. T1	0	0	2	4	0	4	4	0	4
*Ammotium salsum*	0	0	0	4	0	0	0	0	0
***Elphidium oceanense***	296	97	134	188	184	260	288	163	344
*Elphidium selseyense*	28	23	22	16	8	20	94	45	96
***Haynesina germanica***	496	135	202	380	488	416	780	437	676
*Psammophaga* sp.	0	0	0	3	1	0	0	0	0
Total of foraminifera	1384	364	776	1307	1441	1624	1586	694	1492

*Note*: Species in bold are the three most abundant ones.

**FIGURE 7:**
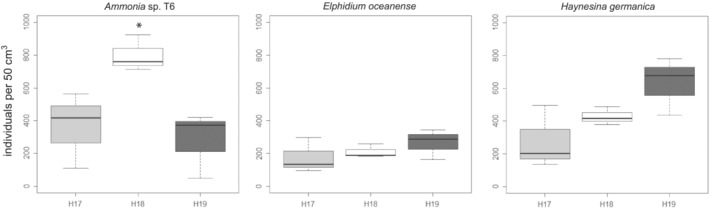
Total densities of the foraminiferal species *Ammonia* sp. T6, *Elphidium oceanense* and *Haynesina germanica* (standardized with numbers of individuals per 50cm^3^) observed in October 2015 for the three stations (H17, H18, and H19). Box plots summed mean of three replicates and standard deviation. A star indicates *Ammonia* sp. T6 density at H18, significantly higher than H17 and H19 densities (Kruskal–Wallis, *p* < .05).

The means of foraminiferal total densities in the stations H17, H18, and H19 are 841 ± 513, 1457 ± 159, and 1257 ± 490 per 50 cm^3^, respectively (Table 3). Although the standard deviation gave an indication of spatial heterogeneity at each station, the densities of foraminifera were not statistically different between the three stations (*n* = 3, Kruskal–Wallis: *H* = 2.49, degree of freedom [df] = 2, *p* = .29). However, the densities of certain species varied between stations. Concerning the most abundant taxa (Figure 7), the density of *Ammonia* sp. T6 was higher in H18 than in other stations (*n* = 3, Kruskal‐Wallis: *H* = 5.6, df = 2, *p* ≤ .05, Dunn's test: H18 > H17, *p* =.03 and H18 > H19, *p* = .01). There was no difference between the three stations for *E. oceanense* (*n* = 3, Kruskal–Wallis: *H* = 1.42, df = 2, *p* = .49). Moreover, although *H. germanica* seemed to be more present in H19, there was no statistical difference with the other stations (*n* = 3, Kruskal–Wallis: *H* = 3.47, df = 2, *p* = .18). The species rankings were the same for H17 and H18 with *Ammonia* sp. T6 being the most abundant, followed by *H. germanica* and *E. oceanense*. For H19, *H. germanica* was the most abundant, followed by *Ammonia* sp. T6 and *E. oceanense*.

### 3.4 Foraminiferal individual and microbiome molecular identification

#### 3.4.1 Individual foraminiferal identification

Specimens H17‐34, H18‐32, and H19‐32 were morphologically and molecularly identified as *Ammonia* sp. T6 (Hayward et al., 2004; Richirt et al., 2019). Specimens H17‐24, H18‐22, and H19‐21 were morphologically identified as *E. oceanense*. H17‐24 and H18‐22 were sequenced and identified as the phylotype S3 (Darling et al., 2016), linked to the morphospecies *E. oceanense*, whereas H19‐21 did not give a positive sequence. Specimens H17‐16, H18‐09, and H19‐10 were morphologically and molecularly identified as *H. germanica* (phylotype S16, Darling et al., 2016).

#### 3.4.2 16S rDNA foraminiferal microbiome identification

**FIGURE 8:**
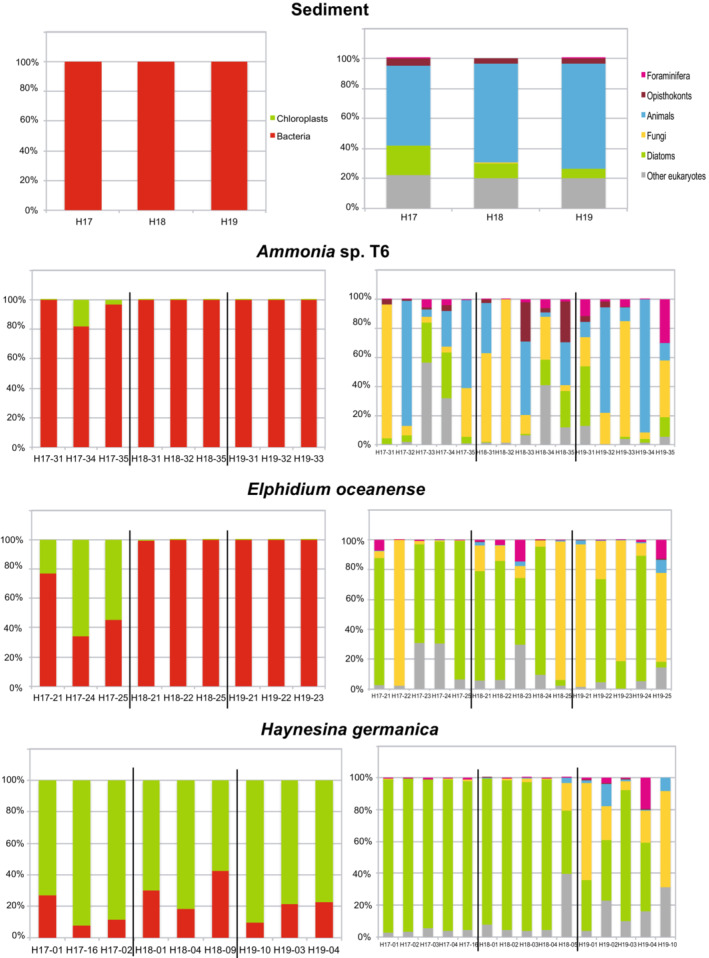
Percentages of HTS reads retrieved from sediment and individuals of the three species for each station (separated by black lines) for 16S (left column) and 18S (right column).

With high‐throughput sequencing, 1,309,496 reads of 16S were obtained from 32 samples (three sediment samples, three specimens for each species and each site, and two negative controls). 935,454 reads corresponding to 29,450 unique sequences and 7963 OTUs reminded after the Mothur analysis. 7159 OTUs with <10 reads representing 1.6% of the reads were removed. In addition, 78 OTUs with 10% or more of the reads sequenced from the negative PCR controls were also removed leaving 756,329 reads and 726 OTUs. Reads belonging to bacteria and chloroplasts were counted from 30 samples, three for sediment and nine for each of the three species (Table 4; Figure 8, 16S). 16S rDNA amplified from sediment was almost exclusively represented by bacterial reads (99.94%–99.99%). 16S from *Haynesina germanica* was mainly from chloroplastic origin (57.89%–92.13%). 16S from *Elphidium oceanense* came from chloroplasts for 22.83%–66.02% in H17, but the percentage of chloroplastic reads was below 1% for H18 and H19. For *Ammonia* sp. T6, only two specimens from H17 had more than 0.5% of chloroplastic reads, the rest of the specimens contained mainly bacterial reads.

**TABLE 4:** Counts of HTS reads for 16S and 18S data per site and per foraminifera

16S			
Sediment	1510H17	1510H18	1510H19
Bacteria	13,685	13,782	15,735
Chloroplasts	2	6	10
Total	13,687	13,788	15,745

*Ammonia* sp. T6	H17‐31	H17‐34	H17‐35	H18‐31	H18‐32	H18‐35	H19‐31	H19‐32	H19‐33
Bacteria	32,221	25,610	22,795	25,023	26,473	20,996	23,024	23,059	20,320
Chloroplasts	29	5730	681	46	12	66	58	6	7
Total	32,250	31,340	23,476	25,069	26,485	21,062	23,082	23,065	20,327

*Elphidium oceanense*	H17‐21	H17‐24	H17‐25	H18‐21	H18‐22	H18‐25	H19‐21	H19‐22	H19‐23
Bacteria	21,417	11,143	13,748	22,489	22,535	19,380	20,478	21,317	27,043
Chloroplasts	6335	21,650	16,546	210	89	25	1	74	82
Total	27,752	32,793	30,294	22,699	22,624	19,405	20,479	21,391	27,125

*Haynesina germanica*	H17‐01	H17‐16	H17‐02	H18‐01	H18‐04	H18‐09	H19‐10	H19‐03	H19‐04
Bacteria	11,757	3295	4770	8366	5025	11,240	2618	1382	5782
Chloroplasts	31,584	38,581	37,935	19,462	22,792	15,453	24,156	5090	19,602
Total	43,341	41,876	42,705	27,828	27,817	26,693	26,774	6472	25,384

18S			
Sediment	1510H17	1510H18	1510H19
Other eukaryotes	19,260	16,195	17,948
Diatoms	17,462	7833	5456
Fungi	135	124	166
Animals	47,006	52,461	61,751
Opisthokonts	4155	2916	2947
Foraminifera	1	0	4
Total	88,019	79,529	88,272

*Ammonia* sp. T6	H17_31	H17_32	H17_33	H17_34	H17_35	H18_31	H18_32	H18_33	H18_34	H18_35	H19_31	H19_32	H19_33	H19_34	H19_35
Other eukaryotes	179	1197	51,704	4283	819	844	1077	4767	25,209	7478	9350	192	2657	1058	4775
Diatoms	3913	3022	25,445	4233	3944	325	137	789	10,989	16,184	29,794	42	811	2192	11,611
Fungi	92,288	4447	3612	545	28,650	44,225	86,051	9536	18,087	2505	14,560	22,802	54,539	3979	34,331
Animals	388	58,267	4797	3246	51,674	24,990	69	37,466	1852	19,069	7370	76,578	6260	77,453	10,387
Opisthokonts	3543	703	1498	524	38	1885	26	20,231	1969	17,847	2902	4279	315	79	40
Foraminifera	2	226	5116	593	673	5	120	1593	3797	1182	8637	1988	3621	238	26,292
	10,0313	67,862	92,172	13,424	85,798	72,274	87,480	74,382	61,903	64,265	72,613	105,881	68,203	84,999	87,436

*Elphidium oceanense*	H17_21	H17_22	H17_23	H17_24	H17_25	H18_21	H18_22	H18_23	H18_24	H18_25	H19_21	H19_22	H19_23	H19_24	H19_25
Other eukaryotes	1692	1053	27,660	23,951	5114	4755	5293	26,641	8733	1887	1060	3601	138	4513	8736
Diatoms	56,596	6	59,591	54,163	74,061	60,324	70,765	39,758	78,454	3810	195	54,552	16,463	71,477	2398
Fungi	3070	46,571	2083	599	441	14,253	9175	7306	3645	85,593	75,526	20,149	71,878	7319	36,407
Animals	163	5	107	277	49	1898	220	2538	248	581	2295	296	152	635	5473
Opisthokonts	0	3	0	0	0	403	256	19	79	89	227	72	21	35	482
Foraminifera	4866	2	557	109	172	955	2898	12,995	181	284	0	323	120	1179	7641
Total	66,387	47,640	89,998	79,099	79,837	82,588	88,607	89,257	91,340	92,244	79,303	78,993	88,772	85,158	61,137

*Haynesina germanica*	H17_01	H17_02	H17_03	H17_04	H17‐16	H18‐01	H18_02	H18_03	H18_04	H18_09	H19_01	H19_02	H19_03	H19_04	H19‐10
Other eukaryotes	1796	1970	4326	3125	3626	5177	3151	3273	4047	38,467	2521	14,738	2941	13,886	26,245
Diatoms	61,751	60,205	74,933	80,384	77,642	63,006	69,960	78,668	90,780	37,921	21,444	24,454	24,210	36,854	11
Fungi	101	59	150	408	844	266	885	2079	203	17,036	40,595	13,628	1549	16,927	50,714
Animals	10	14	25	6	152	107	96	312	273	2987	1167	9285	449	891	6656
Opisthokonts	6	9	1	0	1	13	20	0	8	43	183	145	44	250	0
Foraminifera	407	84	687	443	568	0	142	1	222	3	809	2100	153	16,634	0
Total	64,071	62,341	80,122	84,366	82,833	68,569	74,254	84,333	95,533	96,457	66,719	64,350	29,346	85,442	83,626

For 16S data obtained with the Sanger method, 393 out of 406 sequenced clones gave positive sequences (Table 5). According to public databases BLAST and SILVA ACT, the most numerous sequences belonged to diatom chloroplasts (197 sequences), followed by bacteria (184 sequences), undetermined chloroplasts (four sequences) and embryophyte chloroplast (one sequence). Seven sequences were unclassified (no similarity found). Most of the diatom chloroplastic sequences (77.7%) could not be identified below the phylum level, but a low percentage (22.3%) could be related to seven genera of diatoms: *Gyrosigma* (25 sequences), *Odontella* (seven sequences), *Navicula* (four sequences), *Asterionellopsis* (three sequences), *Lithodesmium* (two sequences), *Pleurosigma* (two sequences), and *Haslea* (one sequence). The identified bacteria were mainly Betaproteobacteria (*Massilia/Oxalobacter*, 162 sequences) and Gammaproteobacteria (*Pseudomonas*, 18 sequences).

**TABLE 5:** Counts of bacterial and chloroplast Sanger sequences (16S rDNA) found for each isolate of foraminifera

	Isolate	Nr clones	Nr sequences	Negative sequences	Unclassified	Bacteria						Chloroplasts		Diatom chloroplasts							
						*Achromobacter*	*Delftia*	*Herminiimonas*	*Massilia/Oxalobacter*	*Pseudomonas*	*Tannerella*	Unknown chloroplast	Embryophyt chloroplast	Unknown diatom chloroplast	*Asterionellopsis* chloroplast	*Gyrosigma* chloroplast	*Haslea* chloroplast	*Lithodesmium* chloroplast	*Navicula* chloroplast	*Odontella* chloroplast	*Pleurosigma* chloroplast
*Ammonia* sp. T6	H1734	48	48			1			25	3				11	1	2				5	
	H1832	33	31	2	1				23	7											
	H1932	50	42	8					41	1											
*Elphidium oceanense*	H1724	48	48		2		1							42				2		1	
	H1822	39	39		1				31	4				3							
	H1921	48	48		2			1	42	3											
*Haynesina germanica*	H1716	48	48		1							1		27	2	11	1		4		1
	H1809	44	42	2							1		1	33		7					
	H1910	48	47	1								3		37		5				1	1
Total		406	393	13	7	1	1	1	162	18	1	4	1	153	3	25	1	2	4	7	2

The percentages of bacterial (potential preys, symbionts, commensals, parasites, or decomposers) or diatom chloroplastic (potential preys or kleptoplasts) sequences varied between foraminiferal species (Table 5). The three replicates of *H. germanica*, a known kleptoplastic species, had a very high percentage of diatom chloroplastic sequences (94%–98%), whereas two out of three replicates of *E. oceanense* and *Ammonia* sp. T6 had a very high percentage of bacterial sequences (90%–100%). The third replicate of *E. oceanense*, H17‐24, harbored 98% of diatom chloroplastic sequences and the third replicate of *Ammonia* sp. T6, H17‐34, 60% of bacterial sequences and 40% of diatom chloroplastic sequences.

#### 3.4.3 18S rDNA foraminiferal microbiome identification

As the chloroplastic 16S sequences cannot be accurately identified at the species or sometimes even the genus level (Pillet et al., 2011), sequenced clones of 18S rDNA were used to refine our identification of the foraminiferal eukaryotic preys and possible kleptoplasts (provided by eaten diatoms), symbionts, parasites, or decomposers.

4,119,961 reads of 18S were obtained from 50 samples (three sediment samples, five specimens for each species and each site, and two negative controls) with high‐throughput sequencing. After the Mothur analysis, 3,845,496 reads corresponding to 70,724 unique sequences and 17,872 OTUs reminded. 15,113 OTUs with <10 reads representing 0.8% of the total reads were removed. In addition, 34 OTUs with 10% or more of the reads sequenced from the negative PCR controls were also removed leaving 3,740,066 reads and 2725 OTUs. Reads belonging to eukaryotic kingdoms or super‐groups were counted for 48 samples, three for sediment and 15 for each of the three species (Table 4; Figure 8, 18S). 18S amplified from sediment was represented by animal reads for more than the half (53.40%–69.96%). The part of diatom DNA was decreasing from almost 20% in H17 to 6% in H19. 18S from *Haynesina germanica* was from diatom origin at more than 90% for all H17 and four H18 samples. For the last H18 sample and three H19 samples, diatomom reads accounted for 30%–40% of the total. The two remaining H19 samples had either 80% or virtually no diatom reads. When diatom DNA was not preponderant, the main source of DNA was either from fungi or other eukaryotes. 18S from *Elphidium oceanense* came from diatoms at more than 2/3 for four samples of H17, three samples of H18 and two samples of H19. Samples where diatom DNA was low had a majority of fungal DNA. For *Ammonia* sp. T6, microbiome taxa were more diversified than for the other species. The dominant DNA either belonged to fungi (four samples) or animalia (five samples), but other eukaryotes or diatoms could also be well represented.

With Sanger sequencing, 262 from the 275 clones selected for 18S gave positive sequences (Table 6). The most numerous sequences belonged to diatoms (191 sequences), followed by fungi (69 sequences) and animalia (two sequences of nematodes). The main sequenced taxa of diatoms were *Thalassiosira* (76 sequences) and *Gyrosigma* (66 sequences), representing >75% of the diatom sequences.

**TABLE 6:** Counts of eukaryotic nuclear Sanger sequences (18S rDNA) found for each isolate of foraminifera

	Isolate	Nr clones	Nr sequences	Negative sequences	Diatoms									Opisthokonts			
					*Ditylum brightwelli*	*Entomoneis* spp.	*Gyrosigma* spp.	*Navicula* sp.	*Nitzschia* spp.	*Odontella* spp.	*Pleurosigma* spp.	*Thalassiosira* spp.	Undetermined diatoms	Uncultured fungi	*Cladosporium* sp.	*Penicillium* sp.	Nematoda
*Ammonia* sp. T6	H1734	48	48	0			3					40	1	2			2
	H1832	29	27	0											1	26	
	H1932	30	27	1											26		
*E. oceanense*	H1724	48	48	0	2	8			2	9		19	8				
	H1822	29	27	0		1		1	6			17				2	
	H1921	13	12	0											12		
*H. germanica*	H1716	48	48	1			47										
	H1809	0	0	0													
	H1910	30	27	0			16				5		6				
Total		275	264	2	2	9	66	1	8	9	3	76	15	2	39	28	2

The percentages of diatoms, fungi, and animalia varied between foraminiferal species; either the fungal or the diatomaceous sequences dominated (Table 6). The two cloned replicates of *H. germanica* had only diatom sequences (100%). The situation was more mixed for the other species. For *E. oceanense*, H17‐24 had 100% of diatomaceous sequences, whereas H18‐22 had 93% of diatomaceous sequences and 7% of fungal sequences, and H19‐21 had 100% of fungal sequences. Among *Ammonia* sp. T6, H17‐34 had 92% of diatom, 4% of fungal, and 4% of animal sequences, while H18‐32 and H19‐32 had 100% of fungal sequences.

**FIGURE 9:**
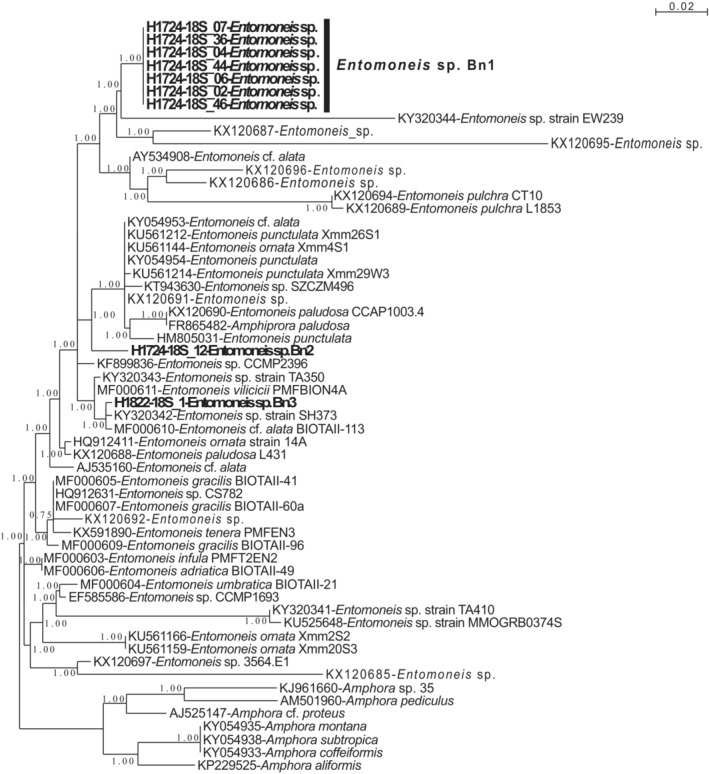
Partial 18S rDNA phylogeny of *Entomoneis* inferred using the ML method with the GTR model and the aLRT SH‐like branch support. Sequences coming from this study are indicated in bold; other sequences come from GenBank. *Amphora* sequences were used as out‐group. 804 out of 866 sites were used and 81.4% of these sites had no polymorphism.

**FIGURE 10:**
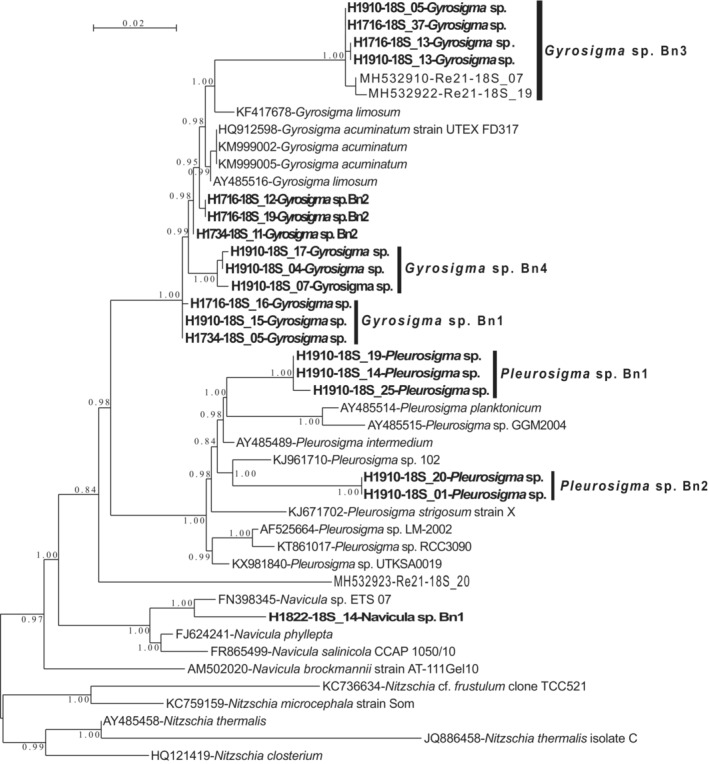
Partial 18S rDNA phylogeny of *Gyrosigma*, *Pleurosigma*, and *Navicula* (Naviculales) inferred using the ML method with the GTR model and the aLRT SH‐like branch support. Sequences coming from this study are indicated in bold; other sequences come from GenBank. *Nitzschia* sequences were used as out‐group. 842 out of 877 sites were used, and 75.4% of these sites had no polymorphism.

**FIGURE 11:**
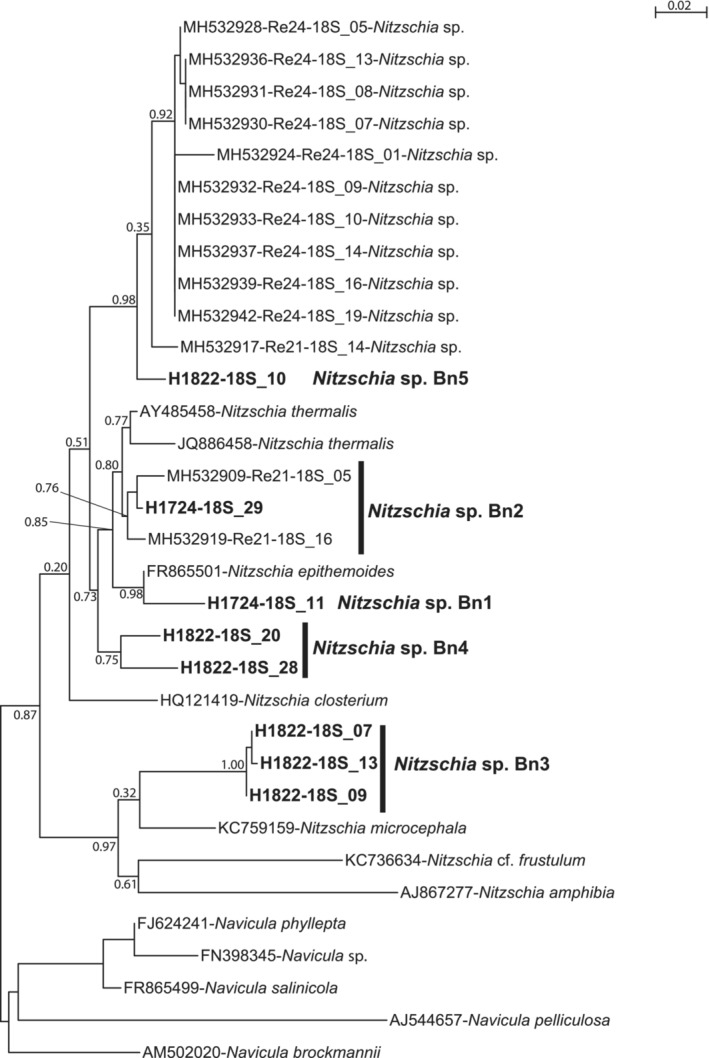
Partial 18S rDNA phylogeny of *Nitzschia* inferred using the ML method with the GTR model and the aLRT SH‐like branch support. Sequences coming from this study are indicated in bold; other sequences come from GenBank. *Navicula* sequences were used as out‐group. 798 out of 871 sites were used, and 81.6% of these sites had no polymorphism.

**FIGURE 12:**
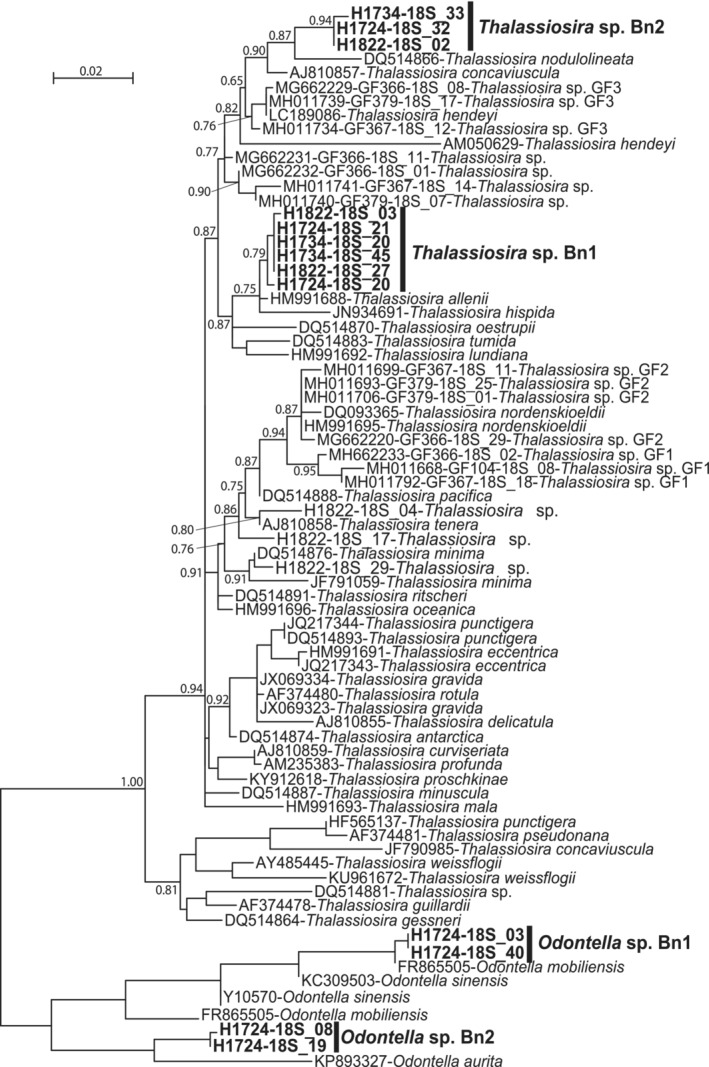
Partial 18S rDNA phylogeny of *Thalassiosira* inferred using the ML method with the GTR model and the aLRT SH‐like branch support. Sequences coming from this study are indicated in bold; other sequences come from GenBank. *Ondontella* sequences were used as out‐group. 799 out of 873 sites were used, and 76.4% of these sites had no polymorphism.

Diatomaceous nuclear sequences belonging to several phylotypes of the same genera were retained for further phylogenetic analyses; four different alignments were made for these subsets and phylogenetic trees were built with these data sets (Figures 9, 10, 11, 12). For *Entomoneis* (Figure 9), three different phylotypes were recognized. For the Naviculales (Figure 10), four phylotypes were recognized for *Gyrosigma*, one for *Navicula*, and two for *Pleurosigma*. Five phylotypes of *Nitzschia* have been identified (Figure 11). The last phylogenetic tree concerned *Thalassiosira* (four phylotypes) and *Odontella* (two phylotypes) (Figure 12). None of these phylotypes were 100% identical to sequences identified at the species level.

#### 3.4.4 Foraminiferal microbiome network analysis

**FIGURE 13:**
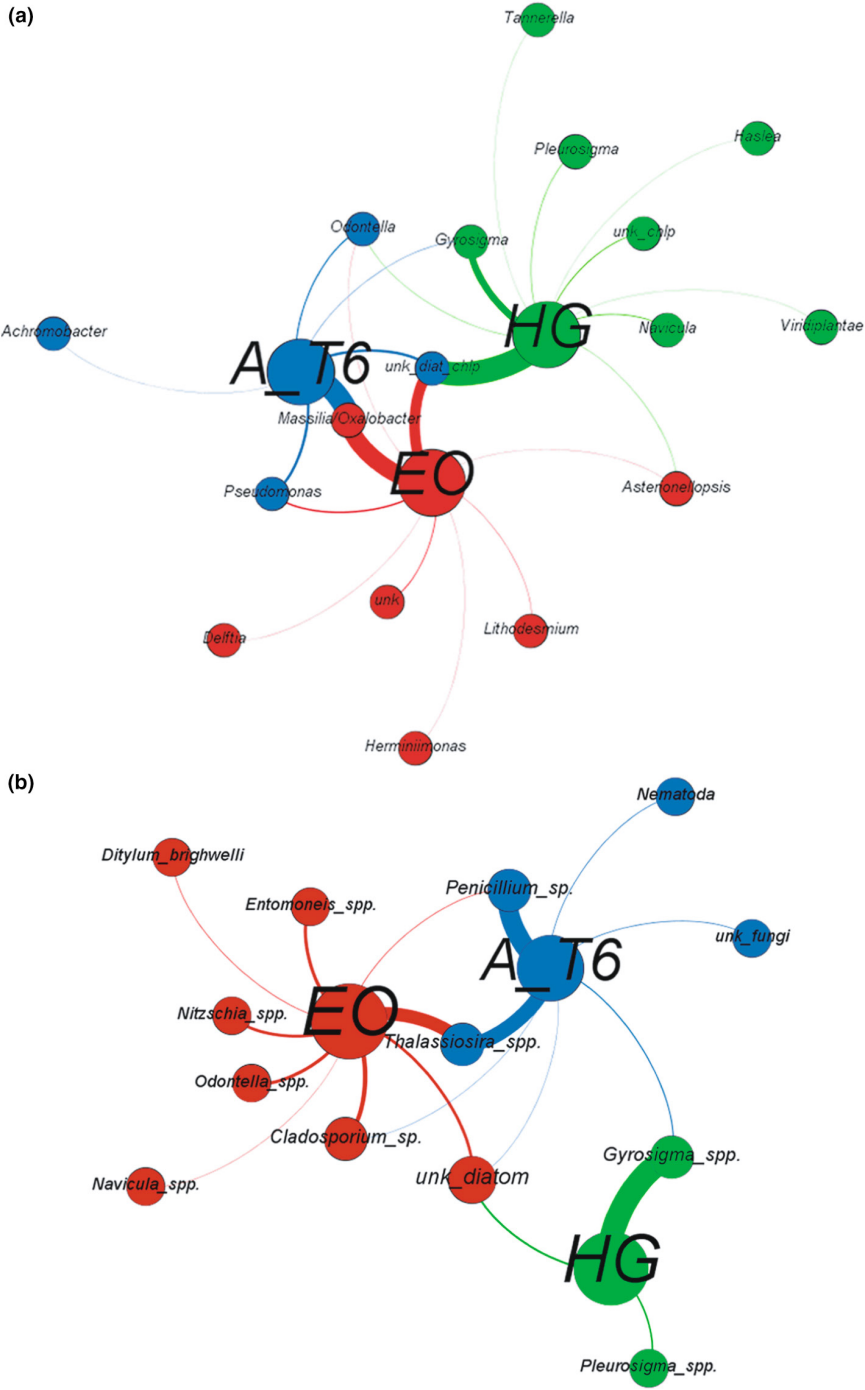
Microbiome network analysis of (a) 16S rDNA and (b) 18S rDNA Sanger sequences extracted from *Ammonia* sp. T6 (A_T6, blue), *Elphidium oceanense* (EO, red), and *Haynesina germanica* (HG, green).

To further investigate the molecular data, a microbiome interspecies comparison was done with DNA network analyses associating rDNA data (16S and 18S) extracted from their respective foraminiferal species and sequenced with the Sanger method (Figure 13). The community analysis with 16S rDNA data highlights microbiome differences for the three studied species (Figure 13a). *Ammonia* sp. T6 contains mainly bacteria and diatoms. Similarly, *E. oceanense* holds bacteria and diatoms, the same bacteria (*Pseudomonas* and *Massilia/Oxalobacter*) and diatoms (*Odontella*) as *Ammonia* sp. T6, and other bacteria (*Herminiimonas* and *Delftia*) and diatoms related to *Asterionellopsis* and *Lithodesmium*. Interestingly, *H. germanica* does not share bacteria in common with the two other foraminiferal species, and bacteria are not driving *H. germanica* abundances as only one bacterial sequence (*Tanneralla* sp.) was detected in one specimen. However, 16S rDNA from different diatoms was identified in *H. germanica*, mostly belonging to benthic pennate diatom genera (e.g., *Gyrosigma*, *Pleurosigma*, and *Navicula*) and more widely distributed genera (*Odontella* and *Asterionellopsis*). As mentioned earlier, the phylogenetic signal of 16S rDNA does not always allow to identify diatoms at the species or even generic level.

The 18S rDNA community analysis based on Sanger sequences also highlights species‐specific microbiomes between the three foraminiferal genera with differences and similarities compared with the 16S rDNA community analysis. *Ammonia* sp. T6 holds diatoms, fungi and nematods. *Elphidium oceanense* contains fungi and diatoms, and its diatom 18S rDNA sequences belong mainly to taxa often encountered in mudflats (e.g., *Nitzschia*, *Entomoneis*, and *Navicula*) and ubiquitous ones such as *Odontella* and *Thalassiosira*. *Haynesina germanica* exclusively retains large pennate diatoms (*Gyrosigma* and *Pleurosigma*) and thus shows a lower taxonomic diversity than the 16S rDNA data.

## 4 DISCUSSION

In tidal mudflats, the species diversity of foraminifera is rather low compared with other environments such as the top of the continental margin (e.g., Bignot, 1985; Fontanier et al., 2002; Mojtahid et al., 2010). In the Bay of Bourgneuf, seven species have been recognized morphologically (see Section 3.3). Among them, the three most common, *Ammonia* sp. T6, *Elphidium oceanense*, and *Haynesia germanica* have been investigated with molecular tools to study their microbiomes. In line with previous studies (Chronopoulou et al., 2019; Salonen et al., 2019), our results show that, despite living in similar habitats, these three species exhibit distinct prokaryotic (16S) and eukaryotic (18S) microbiomes (Figures 8 and 13). These microbiomes are comparatively close to their environmental communities for diatom species (Figure 4, Méléder et al., 2007), but often enriched in fungal DNA and depleted of animal DNA compared with the surrounding sediment (Figure 8). Species‐specific microbiomes also imply that these foraminifera probably have distinct adaptations and possibly diverse trophic strategies. Although these foraminifera are closely related phylogenetically (Schweizer et al., 2008), they evolved in the same environment with different adaptive strategies. This fact is well known for larger organisms such as macrofauna but was never really stated for foraminifera before recently (Chronopoulou et al., 2019; Salonen et al., 2019, 2021).

### 4.1 Densities of MPB, diatoms and foraminifera in stations H17, H18, and H19

NDVI values calculated from satellite data (Figure 1) show that MPB densities are similar between H17 and H18 and decrease in H19. Different grain sizes between stations could explain a change in the diatom assemblages, as large motile epipelic taxa (such as *Gyrosigma* and *Pleurosigma*) tend to decrease or even disappear in sediment with more sand, while small epipsammic species increase (e.g., Hamels et al., 1998; Méléder et al., 2007; Paterson & Hagerthey, 2001; Ribeiro et al., 2013). H17 and H18 are muddy stations (mud >90%) and can be viewed as similar. H19 is considered as sandy mud with less mud than the other stations (mud = 85.6%). The strong presence of *Planothidium delicatulum* in H19 (Figure 4) can be explained by a higher proportion of sand in this station, as this morphospecies is epipsammic. However, the granulometry of this site is not coarse enough to expect an important change in MPB communities (Méléder et al., 2007). Therefore, in the present case, MPB density changes between stations H17‐H18 and H19 can probably not be attributed to grain sizes changes between the three stations. Other factors influencing these site differences could be the distance to the oysters or different currents or interactions with other organisms. For example, the positive feedback of oyster dejections on the microphytobenthos was shown by Méléder et al. (2007) and Echappé et al. (2018).

As MPB is almost only composed of diatoms, it is rather logical that the direct counts of diatoms follow a similar trend as NDVI values, with the highest density in H17 and a decrease in H18 and H19. This is also observed with sediment eDNA data where the percentage of diatoms decreases from H17 to H19 (Figure 8, Sediment 18S). In the three stations, most of the diatoms have a size around 100 μm or lower, but H19 is the only station with no diatom bigger than 100 μm (Figure 5).

**FIGURE 14:**
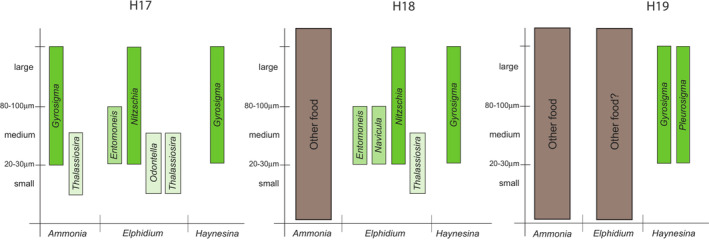
Diagram summarizing the diatomaceous genera and other taxa identified in the microbiome of *Ammonia* sp. T6, *Elphidium oceanense*, and *Haynesina germanica* (Sanger sequencing, 16S and 18S data merged) at the three stations H17, H18, and H19 with a size estimation of the diatoms based on data from Table 2 and Figure 5.

When combining diatoms and foraminifera data (Tables 1, 2, 3, 5 and 6; Figures 5 and 8), we can see that *Ammonia* sp. T6 and *E. oceanense* contain all sizes of diatoms from small to large, whereas *H. germanica* harbors medium to large diatoms from two genera (Figure 14). Moreover, *H. germanica* and *E. oceanense* hold diatoms in stations H17, where diatom density is higher, and H18 (Figures 8 and 14). In station H19, with the lowest density of diatoms and an absence of large diatoms (Table 2, Figure 5), *H. germanica* continues to harbor diatoms with a lower percentage and two specimens of *E. oceanense* still have a majority of diatom DNA (Figures 8 and 14). Station H19 has the lowest diatom density and the highest density of *H. germanica*, which could be explained by a top‐down control, that is, when populations of organisms from lower trophic levels (diatoms here) are controlled by the organisms of higher trophic levels (forams here).

### 4.2 Comparison between morphological and molecular identifications of diatoms

The comparison between morphological (Table 1), 16S (Table 5) and 18S rDNA (Table 6) data to identify diatoms is difficult. Some of the most abundant diatom genera recognized morphologically (*Cocconeis*, *Cymatosira*, *Plagiotropis*, *Planothidium*, and *Staurophora*) were not recognized with DNA datasets (Sanger and HTS). This could either be explained by the absence of these genera in the foraminiferal microbiomes (at least in the most numerous taxa, as only 50 clones were selected for each foraminifer), the taxa selectivity of primers during amplification (primer bias) or possibly by a discrepancy between morphological and molecular taxonomies. Conversely, some of the genera identified with 16S rDNA (*Asterionellopsis*, *Haslea*, and *Lithodesmium*) and 18S rDNA (*Ditylum* and *Entomoneis*) were not recognized with the other datasets. As 16S rDNA has a lower phylogenetic resolution than 18S rDNA (Pillet et al., 2011), different diatom species or genera may share a common 16S rDNA sequence for their chloroplasts. Therefore, previously unsequenced diatoms from Bourgneuf could have the same sequences as *Asterionellopsis*, *Haslea*, and *Lithodesmium*, which could explain why these genera were not retrieved from 18S rDNA and morphological analyses. For 18S rDNA, *Entomoneis* is a diatomaceous genus present in Bourgneuf, but as its frustule is very fragile, it usually disappears during the processes used to prepare the material for morphological observation (see Section 2.2). This fragility could explain the absence of *Entomoneis* from the list of common morphospecies, whereas the genus was recognized with DNA. The absence of *Ditylum* in the morphospecies list could be explained by a discrepancy between morphological and molecular taxonomies or identification problems (e.g., Amato et al., 2007; Kaczmarska et al., 2007).

For the genera identified in both morphological and DNA datasets (*Gyrosigma*, *Navicula*, *Nitzschia*, *Pleurosigma*, *Odontella*, and *Thalassiosira*), there was no species match (Tables 1, 5 and 6; Figures 4 and 9, 10, 11, 12). These results highlight the necessity of dedicated molecular studies to increase the number of species, where 16S rDNA and 18S rDNA are sequenced from the same population and the morphology documented. This would be particularly interesting to increase the number of specimens sampled in the wild.

### 4.3 Possible trophic strategies of three intertidal foraminifera from the Bay of Bourgneuf inferred from their microbiomes

#### 4.3.1 Microbiome and possible trophic strategies of *Ammonia* sp. T6

In H17, *Ammonia* sp. T6 specimens harbored between 80% and 99% of bacterial sequences, whereas specimens from H18 and H19 contained more than 99% of bacterial sequences (Figure 8, 16S). For 18S DNA, there was no clear difference between stations, and some specimens had either a majority of fungal (H17‐31, H18‐31, H18‐32, and H19‐33) or animal (H17‐32, H17‐35, H18‐33, H19‐32, and H19‐34) DNA when others had more even distributions (H17‐33, H17‐34, H18‐34, H18‐35, H19‐31, and H19‐35) (Figure 8, 18S). H18 is the station with highest densities of *Ammonia* sp. T6 densities compared with H17 and H19. Station H17 has the highest density of diatoms, and this value is strongly decreasing in H18 and H19 (Table 1), but this decrease is not observed in *Ammonia* sp. T6 microbiomes sequenced with HTS (9%–12% of diatom reads). In addition, the diatoms caught by this foraminifer have small to large sizes (Figure 14).

These results agree well with what is known on *Ammonia* in the literature. This genus is thought to be omnivorous, feeding on organic detritus, bacteria, microalgae, and meiofauna such as nematods (Dupuy et al., 2010; Mojtahid et al., 2011; Pascal et al., 2009; Wukovits et al., 2018). A study using a metabarcoding approach with 18S rDNA confirmed the omnivorous diet of *Ammonia* sp. T6, composed mainly of diatoms and meiofaunal metazoans with large percentage variations between individuals (Chronopoulou et al., 2019). Several ultrastructural studies have shown the total ingestion of diatom frustules by *Ammonia* sp. (Jauffrais et al., 2018; LeKieffre et al., 2017). In addition, diatom chloroplasts quickly become nonfunctional in this taxon as they are digested, demonstrating that this foraminifer is not kleptoplast (Jauffrais et al., 2016, 2018). Moreover, bacteria are preyed on by *Ammonia* under oxic conditions (Pascal et al., 2008), but could be symbionts under anoxic conditions (Koho et al., 2018; Nomaki et al., 2014; Salonen et al., 2019).

Comparing our results and the literature, *Ammonia* sp. T6 can thus be described as a heterotrophic omnivorous foraminifer with different trophic strategies depending on resources availability. It feeds on bacteria, animalia, diatoms, other eukaryotes, and maybe fungi (Figures 8 and 13). Nevertheless, further studies are needed to check whether *Ammonia* sp. T6 is actively hunting and/or scavenging metazoans in natural conditions and to investigate the role of fungi (preys, commensals, decomposers, and parasites?). Moreover, an accurate identification of the *Ammonia* species is needed, as the morphospecies *Ammonia tepida*, often identified in ecological studies, is represented by three different phylotypes in Europe: T1, T2, and T6 (Bird et al., 2020; Hayward et al., 2004), which can now be distinguished morphologically (Richirt et al., 2019). The present study and the ones of Chronopoulou et al. (2019), Jauffrais et al. (2016, 2018) and LeKieffre et al. (2017) deal with *Ammonia* sp. T6, but *Ammonia* sp. T1 and T2 could have different trophic behaviors.

#### 4.3.2 Microbiome and possible trophic strategies of *Elphidium oceanense*

With 16S data, the specimens of *E. oceanense* from H17 had the highest percentage of chloroplastic sequences with 22%–66%, whereas specimens from H18 and H19 had more than 99% of bacterial DNA (Figure 8, 16S). It was more contrasted for 18S data, ten specimens had a majority of diatomaceous sequences (44%–92% of the total) and the five remaining specimens had a majority of fungal sequences (80%–98% of the total) (Figure 8, 18S). H19‐21 had 100% of bacterial sequences and 95.2% of fungal sequences (Figure 8). As no foraminiferal DNA could be amplified from this replicate, it may have been dead at the time of collection (see Schweizer, 2015) and its microbiome would be the reflection of the decay mechanisms happening after its death with bacteria and fungi acting as decomposers. The same may be true for specimens H17‐22, H18‐25, and H19‐25, which also contained a majority of fungal DNA (no data for 16S). The densities of *E. oceanense* are similar in the three stations, and this is always the less abundant of the three main foraminiferal species (Table 3, Figure 7). The diatoms caught by this foraminifer have small to large sizes and a higher taxonomic diversity than in *Ammonia* and *Haynesina* (Figure 14). The microbiome network analysis showed that *E. oceanense* contained bacteria, diatoms, and fungi, some similar to *Ammonia* sp. T6 ones, and others (bacteria *Herminiimonas* and *Delftia*, diatoms *Nitzschia*, *Entomoneis*, *Navicula*, and *Odontella*) not shared by other foraminifera (Figure 13). *Herminiimonas* was isolated only from H19‐21, and as this specimen could have been dead at the time of collection, the bacterium could be linked to decay processes.

It is very difficult to find information on *E. oceanense* in the literature, as this species was often mixed with other ones in the *E. excavatum* morphospecies. Comparisons in this group are difficult. For example, *Elphidium selseyense*, which was also included in the *E. excavatum* group, is a kleptoplastic species (Chronopoulou et al., 2019; Jauffrais et al., 2018). However, *E. oceanense* is different morphologically, genetically, and physiologically from *E. selseyense* (Darling et al., 2016; Jauffrais et al., 2018). There is only one ultrastructural study with a clear identification of *E. oceanense* where the ingested chloroplasts are in degraded states (Jauffrais et al., 2018), showing that they are eaten, but probably not used for photosynthesis. Unpublished data (T. Jauffrais) showed that it has no functional kleptoplasts (maximum photosynthetic efficiency [Fv/Fm] = 0 and no oxygenic photosynthesis). Bacteria and fungi in *E. oceanense* microbiome could be either preys, symbionts, commensals, and parasites (or decomposers for H19‐21, which was probably dead at the time of collection) and further studies including Transmitted Electronic Microscopy (TEM) would be needed to investigate their roles.

Therefore, we hypothesize that *E. oceanense* is a probable heterotrophic herbivorous foraminifer feeding mainly on diatoms (Figures 8 and 13). Where diatoms are less abundant (e.g., H19), *E. oceanense* possibly mixes diatoms with other preys (bacteria and fungi, unless they have a different role; Figures 8 and 13).

#### 4.3.3 Microbiome and possible trophic strategies of *Haynesina germanica*

The replicates of *H. germanica* had a majority (57.9%–92.1%) of chloroplastic sequences in all stations, contrary to other foraminiferal species (Figure 8, 16S). All specimens from H17 and four specimens from H18 had more than 90% of diatom nuclear sequences, whereas in the remaining specimens, diatom sequences were either still in majority (H19‐02, H19‐03, and H19‐04) or equal with other eukaryotes (H18‐09), or fungal sequences were prominent (H19‐01 and H19‐10) (Figure 8, 18S). Although diatom densities are decreasing from H17 to H19 (Table 1), *H. germanica* is equally present in the three stations (Table 3, Figure 7) and is relatively more abundant than *Ammonia* sp. T6 in H19, the station with the lowest diatom density. As nuclear DNA is not supposed to be retained by kleptoplastic species, the 18S rDNA may reflect the recent feeding activity of *H. germanica* contrary to chloroplastic DNA. The discrepancy with 16S rDNA sequence identification could also be explained by the low phylogenetic resolution of 16S data, either short or long amplicon (Figure 13a vs. b). With the Sanger method, only one bacterial sequence was sampled in *H. germanica*, and it is not shared by the two other foraminiferal species, other 16S rDNA sequences were identified as benthic pennate diatoms (*Gyrosigma*, *Pleurosigma*, and *Navicula*) and other less well‐identified taxa (Table 5, Figure 13a). Our data show a clear preference of *H. germanica* for diatoms among other preys (Figure 8, 18S). The diatoms caught by this foraminifer have medium to large sizes (Figure 14), even when these taxa/sizes are scarce or virtually absent in the environment (Tables 1 and 2, Figure 5).

*Haynesina germanica* has been shown to crack frustules of large diatoms and suck out the cell content while keeping the frustule outside their shell (Austin et al., 2005; Jesus et al., 2021). Moreover, it is known to be kleptoplast‐bearing, which means able to steal chloroplasts from its diatom preys and use them to perform photosynthesis (Jauffrais et al., 2016, 2018; Jesus et al., 2021; LeKieffre et al., 2018; Lopez, 1979). Studies using a metabarcoding approach confirmed the specialized diatom diet of *Haynesina germanica* (16S rDNA: Pillet et al., 2011; 18S rDNA: Chronopoulou et al., 2019).

In light of our data and the literature, *H. germanica* can be described as a mixotrophic herbivorous foraminifer, specialized in medium‐large pennate diatom preys and performing kleptoplasty. It is hunting these diatoms even when they are not abundant in the environment (Table 1, Figures 5 and 14). Station H19 has the lowest diatom density and the highest density of *H. germanica*, which could translate a top‐down control. It is less clear in stations H17 and H18, but *Haynesina* may control the structure and population dynamics of MPB (mainly composed of diatoms) by eating selectively the largest diatoms in all stations. The diet of *H. germanica* is therefore more specialized than the one of *Ammonia* sp. T6 and *E. oceanense*. Contrary to *Ammonia* sp. T6, which phagocytizes whole diatoms with their frustules (Jauffrais et al., 2018; LeKieffre et al., 2017), *H. germanica* maintains the frustule with its reticulopods and only sucks the diatom cell content without engulfing the frustule (Austin et al., 2005). This shows that foraminifera living in the same habitats may use different ways to feed on the same kind of preys (diatoms). There are discussions about the presence/absence of diatom nuclei in kleptoplastic foraminifera (e.g., Jauffrais, LeKieffre, Schweizer, Geslin, et al., 2019; Jauffrais, LeKieffre, Schweizer, Jesus, et al., 2019; Pillet et al., 2011), but as most of the photosynthesis genes are in the nucleus instead of the chloroplast (Eberhard et al., 2008), the diatom nucleus may be needed in the foraminiferal cytoplasm to keep the kleptoplasts running. As *H. germanica* is probably continually feeding on diatoms in its natural environment, diatom nuclei could always be present in its cytoplasm. Our 18S rDNA data and the ones of Chronopoulou et al. (2019), with a more stringent cleaning procedure, show the presence of very pristine diatom nuclear DNA in *H. germanica* (as DNA sequencing can only succeed with intact nucleic acids). However, further studies with nuclear staining and TEM would be needed to confirm the presence of healthy diatom nuclei in kleptoplastic foraminifera.

## 5 CONCLUSION

Our results provide new information about foraminiferal ecology with an original combination of molecular and morphological data of foraminifera and diatoms, revealing the complex interactions between these protists through symbiosis or trophic relationships. To summarize, three different trophic strategies can be deduced for the foraminiferal species investigated here. *Ammonia* sp. T6 is a heterotrophic omnivorous foraminifer with different trophic strategies depending on resources availability and is feeding on diatoms only when they are very abundant. *Elphidium oceanense* is a probable heterotrophic herbivorous foraminifer, preferably feeding on diatoms when they are abundant. *Haynesina germanica* is a mixotrophic herbivorous foraminifer, specialized in medium to large pennate diatom preys and performing kleptoplasty. We can conclude that *Ammonia* sp. T6 is probably more opportunistic than *E. oceanense* and *H. germanica* (Figures 8, 13 and 14), with a wider diet as it can even prey on nematods (Dupuy et al., 2010) and other metazoa (Chronopoulou et al., 2019).

This study, together with other recent ones on foraminifera (Chronopoulou et al., 2019; Jauffrais, LeKieffre, Schweizer, Geslin, et al., 2019; Jauffrais, LeKieffre, Schweizer, Jesus, et al., 2019; Pillet et al., 2011; Prazeres et al., 2017; Schmidt et al., 2016; Tsuchiya et al., 2015), highlights the importance of molecular tools to study trophic interactions and microbiome communities of protists at the single‐cell scale. In addition, this study shows the relevance of combining molecular and morphological tools for studying trophic interactions and relationships between protists and their microbial associates using single‐cell analysis and morphological counting methods to assess densities. Nevertheless, as mentioned earlier, some limitations linked to the lack of data in DNA databases and to the difficulty to compare morphological and molecular data may be noticed. These limitations will require further dedicated studies to be able to tackle the holobiont of single‐cell eukaryotes with a higher accuracy. Moreover, additional studies focusing more on metazoa, fungi, and bacteria in the environment and in the foraminifera, using labeled organic matter and/or investigating the ultrastructure of foraminifera, are needed to go further.

## AUTHOR CONTRIBUTIONS

**Magali Schweizer:** Conceptualization (lead); data curation (equal); formal analysis (equal); writing – original draft (lead); writing – review and editing (lead). **Thierry Jauffrais:** Conceptualization (equal); formal analysis (equal); writing – original draft (equal); writing – review and editing (supporting). **Constance Choquel:** Conceptualization (equal); data curation (equal); formal analysis (equal); writing – original draft (supporting); writing – review and editing (supporting). **Vona Méléder:** Conceptualization (equal); data curation (equal); formal analysis (equal); writing – original draft (supporting); writing – review and editing (supporting). **Sophie Quinchard:** Data curation (supporting); formal analysis (supporting); writing – original draft (supporting); writing – review and editing (supporting). **Emmanuelle Geslin:** Conceptualization (equal); data curation (supporting); formal analysis (supporting); funding acquisition (lead); project administration (lead); writing – original draft (supporting); writing – review and editing (supporting).

## CONFLICT OF INTEREST

We have no conflict of interest.

## Data Availability

HTS reads have been deposited in the Sequence Read Archive (SRA) at NCBI under accession number PRJNA803142 (https://www.ncbi.nlm.nih.gov/sra/PRJNA803142). Sanger sequences have been submitted to the DDBJ/EMBL/GenBank databases under accession numbers MH748140‐MH748142, MH754956‐MH755215, MH817163‐MH817344, and MH818857‐MH819051. KY347799 and KY347800 were previously published in Jauffrais et al. (2018).
